# Rac1 controls cell turnover and reversibility of the involution process in postpartum mammary glands

**DOI:** 10.1371/journal.pbio.3001583

**Published:** 2023-01-19

**Authors:** Aleksandr Mironov, Matthew Fisher, Priya Narayanan, Randa Elsayed, Melis Karabulutoglu, Nasreen Akhtar

**Affiliations:** 1 Faculty of Biology Medicine and Health, University of Manchester, Manchester, United Kingdom; 2 The Bateson Centre and Department of Oncology and Metabolism, University of Sheffield, Sheffield, United Kingdom; Consejo Nacional de Investigaciones Científicas y Técnicas: Consejo Nacional de Investigaciones Cientificas y Tecnicas, ARGENTINA

## Abstract

Cell turnover in adult tissues is essential for maintaining tissue homeostasis over a life span and for inducing the morphological changes associated with the reproductive cycle. However, the underlying mechanisms that coordinate the balance of cell death and proliferation remain unsolved. Using the mammary gland, we have discovered that Rac1 acts as a nexus to control cell turnover. Postlactational tissue regression is characterised by the death of milk secreting alveoli, but the process is reversible within the first 48 h if feeding recommences. In mice lacking epithelial Rac1, alveolar regression was delayed. This defect did not result from failed cell death but rather increased cell turnover. Fitter progenitor cells inappropriately divided, regenerating the alveoli, but cell death also concomitantly accelerated. We discovered that progenitor cell hyperproliferation was linked to nonautonomous effects of Rac1 deletion on the macrophageal niche with heightened inflammation. Moreover, loss of Rac1 impaired cell death with autophagy but switched the cell death route to apoptosis. Finally, mammary gland reversibility failed in the absence of Rac1 as the alveoli failed to recommence lactation upon resuckling.

## Introduction

Cell turnover in adult tissues is characterised by the death of older cells and replacement with new through stem and progenitor cell proliferation. How these processes are balanced to maintain long-term tissue homeostasis is not clearly understood. The mammary gland is an example of a tissue that maintains a state of flux throughout the adult life. It also undergoes periods of profound growth and regression in each reproductive cycle, providing a tractable model to study cell turnover. The primary role of the mammary gland is to produce milk as a source of nutrients to feed the newborn. Successful lactation depends on the coordinated development and differentiation of the secretory alveolar epithelium during pregnancy and the subsequent removal of these milk-producing units once the milk supply is no longer needed. The balance of cell death and cell division continuously alters to permit tissue growth and regression within the mammary gland reproductive cycle, but little is known about how this is coordinated.

Weaning of the infants triggers the mammary gland to enter postlactational involution, a process in which the surplus milk secreting alveolar epithelium is pruned from the ductal tree using a controlled cell death program [1]. In rodents, 90% of the alveolar epithelium laid down in pregnancy is subsequently removed during involution, with the balance of cell turnover tipped towards cell death and the remodelling process completed within approximately two weeks. In murine models of forced involution, simultaneous weaning of the pups at the peak of lactation causes the secretory alveoli to become engorged with milk as production continues for the first 24 h, after which they dedifferentiate. Both mechanical stretch and milk factors have been reported to stimulate cell death [1,2]. A number of programmed cell death mechanisms have been identified in postlactational involution, including apoptosis, lysosomal permeabilisation, and cell death with autophagy, although the significance of the different death pathways is unclear [3–7]. Moreover, lysosomal permeabilisation and autophagy may feed into an apoptotic death downstream. Involution occurs in two phases; In the first 48 h, cell death is triggered but the process of involution is reversible and the gland can reinitiate lactation once suckling resumes [3,8]. The second phase is irreversible and is characterised by destruction of the subtending basement membrane, extensive alveolar cell death, and repopulation of stromal adipocytes. The dead cells and residual milk are primarily removed by neighbouring live alveolar mammary epithelial cells (MECs) that act as phagocytes [9–12]. Engulfment of milk fat by the nonprofessional MECs triggers lysosomal permeabilisation through a stat-3-dependent mechanism, which ultimately kills the cells [12]. In the second phase, professional phagocytes from the immune system enter the gland to engulf the remaining dead cells and the tissue remodels back to a state closely resembling the nulliparous gland [13,14].

We previously showed that the Rac1 GTPase plays a crucial role in postlactational mammary gland remodelling [9]. Rac1 is central to the conversion of MECs into nonprofessional phagocytes for the removal of dead cells and surplus milk and in controlling the inflammatory signature. We now reveal further roles for Rac1 in controlling the involution process. We have discovered that Rac1 acts as a nexus, controlling both the rate and balance of cell death and progenitor cell division in involution. Without Rac1, cell turnover accelerates with consequences on mammary gland remodelling in the irreversible phase. Moreover, failure to redifferentiate blocks mammary gland reversibility in the first phase of involution. Rac1 therefore has multifaceted roles in orchestrating the involution process.

## Results

### Delayed alveolar regression and repopulation of adipocytes in involuting *Rac1−/−* mammary glands

**Fig 1:**
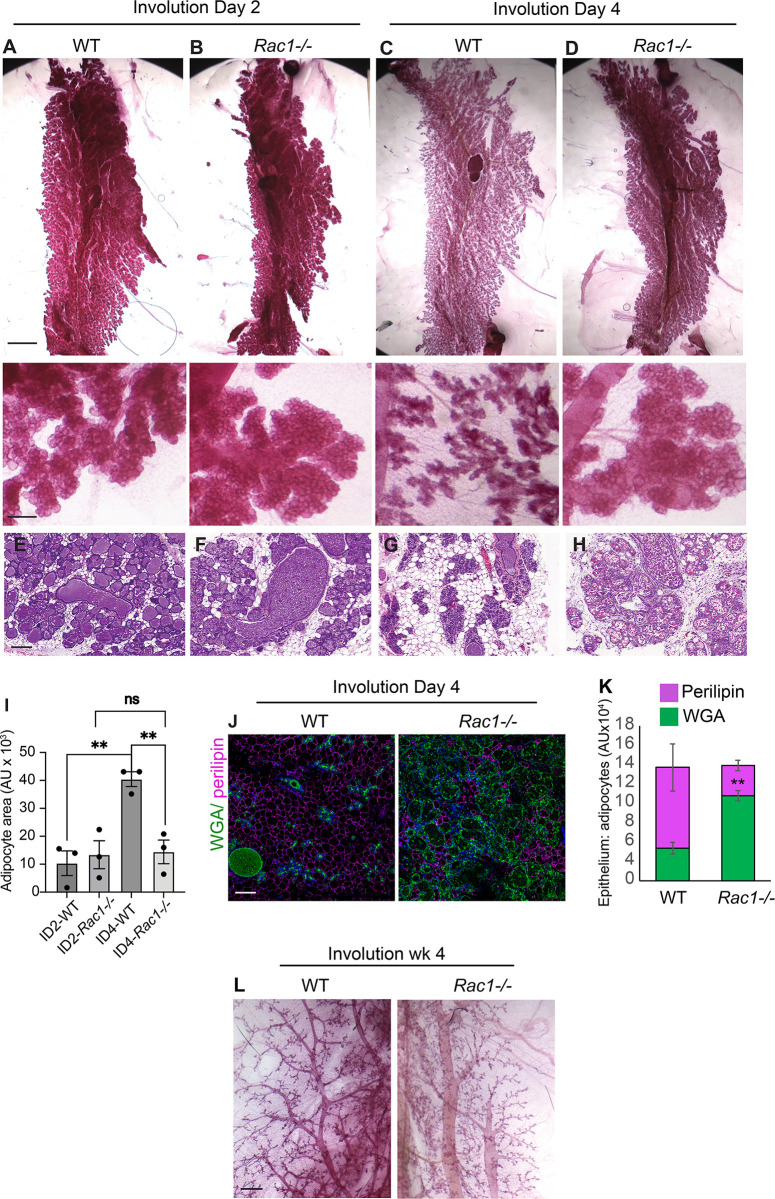
Delayed alveolar regression in *Rac1−/−* glands in involution. (**A**-**D**) Carmine staining of whole-mounted mammary glands of WT (**A**, **C**) and *Rac1−/−* (**B**, **D**) mice at postlactational involution day 2 and day 4. Note the alveolar regression in WT but not *Rac1−/−* glands at involution day 4. Bar: 5 mm, (inset 1 mm). (**E**-**H**) Haematoxylin–eosin (HE) stain shows alveolar regression and adipocyte repopulation in WT glands (**E**, **G**), but this is delayed in *Rac1−/−* glands (**F**, **H**). Bar: 100 μm. (**I**) Quantification of adipocyte repopulation in HE images. Error bars: +/− SEM of *n =* 3 mice per group. ** *P* ≤ 0.01. (**J**) Alveolar regression and adipocyte repopulation at involution day 4 were confirmed by immunofluorescence staining with WGA-488 (green) to detect epithelium and perilipin antibody (magenta) to detect adipocytes. Bar: 100 μm. (**K**) Quantification of alveolar regression and adipocyte repopulation. Error bars: +/− SEM of *n* = 3 mice per group. ** *P* ≤ 0.01. (**J**) Carmine staining of whole-mounted WT and *Rac1−/−* mammary glands at 4 weeks postlactational involution show complete regression of alveoli. Note: Bloated ducts persist in *Rac1−/−* glands. Bar: 0.7 mm. The data underlying the graphs shown in this figure can be found in S1 Data.

Mammary gland remodelling in involution is accompanied by alveolar regression and concomitant fat pad repopulation in the surrounding parenchyma. To investigate the role of Rac1 in tissue regression during involution, we examined mammary tissues from female mice triggered to involute through simultaneous weaning of the pups. The Rac1 gene was deleted specifically in luminal cells of the mammary gland using *Rac1*^*fl/fl*^:*LSLYFP*:*WAPiCre* (*Rac1−/−*) conditional knockout mice previously generated [15]. Cre-negative *Rac1*^*fl/fl*^:*LSLYFP* littermates were used as wildtype (WT) controls. Analysis of the WT mammary glands by histology and immunofluorescence staining of epithelial and adipocyte markers revealed significant lobular alveolar regression and adipocyte repopulation between involution days 2 to 4 (Figs 1A, 1C, 1E, 1G, 1I, 1J, 1K and S1). By contrast, alveoli in *Rac1−/−* glands remained distended with virtually no adipocyte repopulation (Figs 1B, 1D, 1F, 1H, 1K and S1). Four weeks postinvolution, the alveoli had completely regressed in both WT and *Rac1−/−* glands, confirming that lobular alveolar cell death had occurred in the absence of Rac1 (Fig 1L).

### β1-integrin is not upstream of Rac1 in alveolar regression

**Fig 2:**
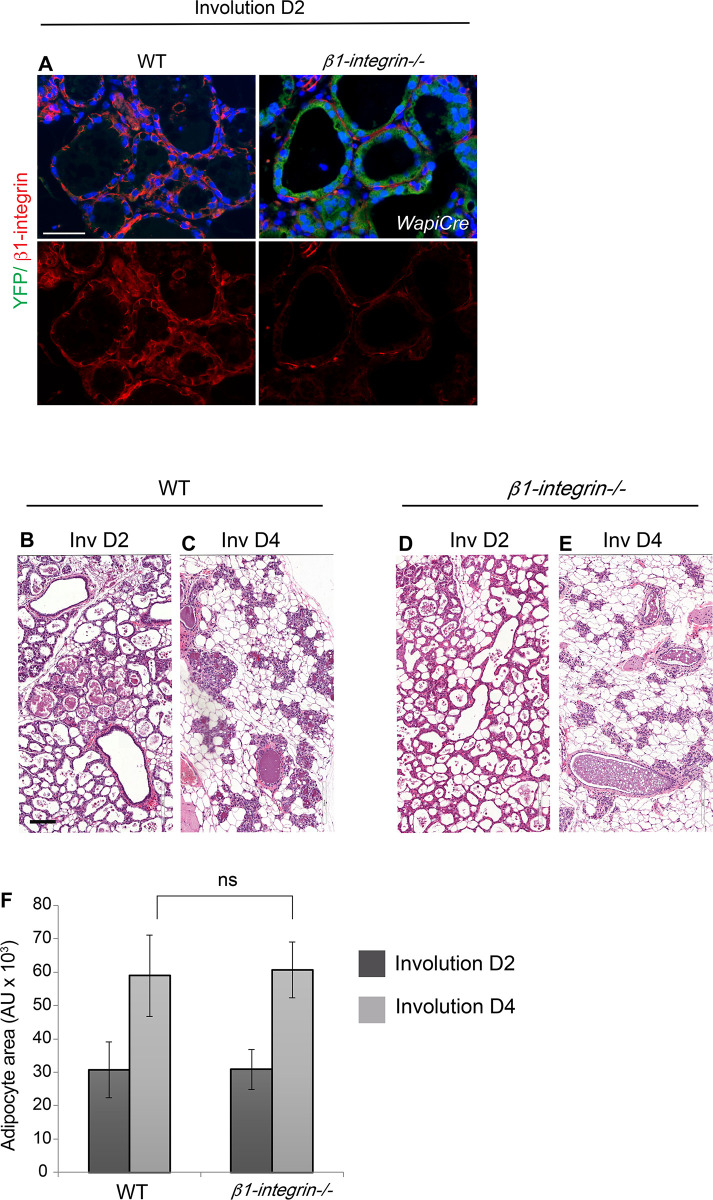
Ablation of β1-integrin does not phenocopy the *Rac1−/−* involution phenotype. (**A**) Ablation of β1-integrin in luminal cells at involution day 2 was detected by immunofluorescence staining with a β1-integrin antibody (red). YFP reporter gene expression (green) confirmed Cre-mediated recombination in transgenics. Bar: 50 μm. (**B**-**E**) HE stains at involution days 2 and 4 show no delay in alveolar regression in *β1-integrin−/−* mice. Both WT glands (**A**, **B**) and *β1-integrin−/−* (**C**, **D**) glands equally regressed. Bar: 100 μm. (**E**) Quantification of adipocyte repopulation. Error bars: +/− SEM of *n =* 4 mice per group. NS (not significant). The data underlying the graphs shown in this figure can be found in S1 Data.

In mammary gland tissue, β1-integrin functions upstream of Rac1 to regulate lactational differentiation, stem cell renewal, and cell cycle progression [9,16–19]; we thus investigated whether β1-integrin was linked to alveolar regression in involution. The β1-integrin gene was deleted in luminal cells of the mammary gland using *β1-integrin*^*fl/fl*^:*LSLYFP*:*WAPiCre* conditional knockout mice (Fig 2A). Crucially, tissue analysis at involution days 2 and 4 revealed that loss of β1-integrin did not impair alveolar regression and adipocyte repopulation compared to WT mice of a Cre-negative genotype (*β1-integrin*^*fl/fl*^:*LSLYFP*; Fig 2B–2F). This indicates that Rac1’s role in tissue regression is elicited through a distinct upstream signalling axis.

### Rac1 ablation imbalances cell turnover rates in involution causing delayed alveolar regression

**Fig 3:**
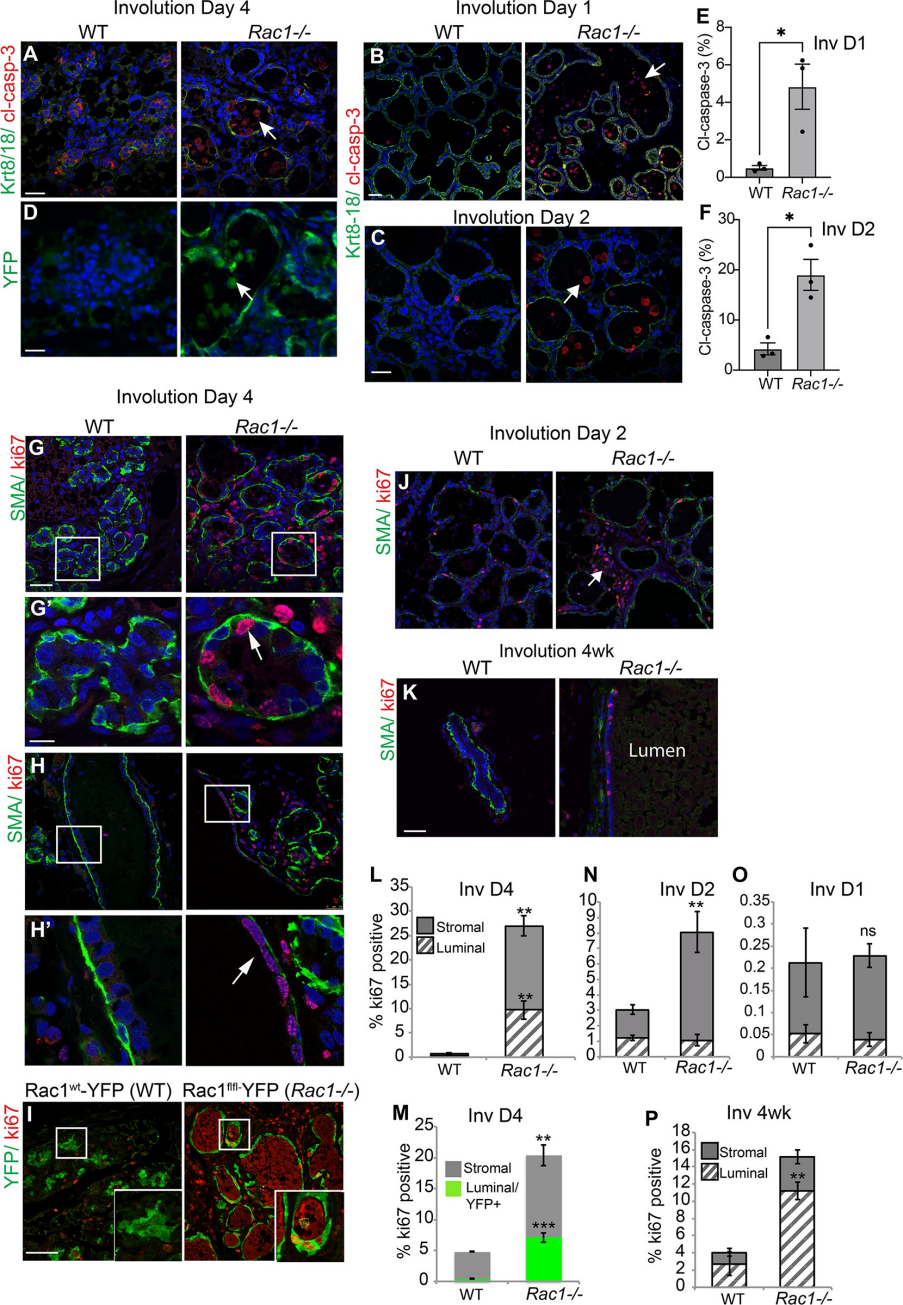
Delayed alveolar regression is not due to impaired cell death but heightened proliferation. (**A**-**C**) Immunofluorescence staining with cleaved caspase-3 (red) antibody shows numerous dead cells in *Rac1−/−* alveolar lumens at involution day 4 (**A**) and involution days 1 (**B**) and 2 (**C**). Keratin (Krt) 8/18 (green) was used to detect luminal cells. Note the Krt8-18 antibody cross-reacts with intact keratins in live cells and not cleaved forms in dead cells. Arrows: caspase-positive dead cells in the lumen. Bar: 40 μm. (**D**) Dead cells in the lumen were confirmed to be of luminal origin with the YFP reporter gene expression. GFP antibody was used to stain the YFP reporter gene. Arrowhead: dead cells in the lumen. Bar: 20 μm. (**E**, **F**) Quantification of cleaved caspase-3-positive cells at involution days 1 (**E**) and 2 (**F**). Error bars: +/− SEM of *n =* 3 mice per group. * *P* ≤ 0.05. (**G**, **H**) Immunofluorescence staining for proliferation marker Ki67 (red) reveals increased proliferation within *Rac1−/−* glands at involution day 4 in alveoli (**G**, **G’**) and ducts (**H**, **H’**). **G’**, **H’** are zoomed images. Arrows point to ki67-positive luminal cells in alveoli and ducts. Epithelial tissue boundary was detected by smooth muscle actin (SMA, green) present in myoepithelial cells. Bar: 50 μm. (**I**) Proliferating cells were confirmed to be of luminal origin with the YFP reporter gene expression. *Rac1*^*wt*^:*WAPiCre*:*YFP* mice were used as WT controls to directly compare YFP-positive luminal cells. Ki67 antibody was used to detect proliferation. (**J**, **K**) Ki67 staining in mammary glands at day 2 and week 4 postlactational involution. Note: Proliferation persists in bloated *Rac1−/−* ducts at 4 weeks. Bar: 50 μm. (**L**-**P**) Quantitative analysis of Ki67 at involution day 4 (**L**, **M**), day 2 (**N**), day 1 (**O**), and week 4 (**P**). Proliferating cells were scored as either luminal or stromal/fat pad. SMA was used to count cells within the epithelial boundary (**L**, **N**-**P**). Luminal cell proliferation was confirmed by scoring YFP+/Ki67+ cells. Error bars: +/− SEM of *n =* 3 mice per group. *** *P* ≤ 0.001, ** *P* ≤ 0.01, ns; not significant. The data underlying the graphs shown in this figure can be found in S1 Data.

We first investigated whether there was an initial delay in cell death in *Rac1−/−* glands, which might explain the delay in alveolar regression. At involution day 4 where the delayed regression is most prominent, numerous cell corpses were evident in *Rac1−/−* glands both within the alveolar epithelium and within the lumen space (S2A Fig). We confirmed the dead cells with cleaved caspase-3 staining and that they were of luminal cell origin with the *Rosa*:*LSL-YFP* reporter gene, which is activated in response to *WAPiCre*-induced recombination (Fig 3A and 3D). Moreover, cell death was not delayed within the earlier reversible involution stage (days 1 and 2), but rather increased cell corpses were detected in *Rac1−/−* tissue lumens (Figs 3B, 3C, 3E, 3F, S2A and S2B). However, this might be a result of defective clearance by epithelial phagocytes as opposed to increased cell death in *Rac1−/−* glands [9]. Thus, the delay in alveolar regression in transgenic glands is not linked to impaired cell death.

Retention of milk within the lumens might contribute to the distended alveolar phenotype in *Rac1−/−* glands, since Rac1 is crucial for epithelial cell-directed engulfment of apoptotic cell corpses and residual milk [9]. However, given the extensive cell death detected in early involution, it was surprising that the alveoli remained intact. We thus investigated whether *Rac1−/−* alveoli were being maintained through cell renewal. Ki67 staining revealed almost no detectable proliferation in WT glands at involution day 4. In contrast, *Rac1−/−* mammary glands showed extensive proliferation within duct and alveolar luminal cells and within cells in the surrounding stromal/fat pad areas (Figs 3G, 3H, 3L and S2C). To confirm that proliferation was occurring within *Rac1−/−* luminal cells and not from cells that had escaped recombination, we scored proliferation in YFP-positive/*Rac1−/−* cells using the *Rosa*:*LSL*:*YFP* reporter gene (Fig 3I and 3M) Proliferation within the transgenic ducts continued at 4 weeks postinvolution; at this stage, however, most of the lobular alveoli had regressed (Figs 1L, 3K and 3P). Analysis of the phase I-reversible stage of involution (days 1 and 2) revealed no significant difference and very little proliferation at day 1. In contrast, at involution day 2, increased proliferation was detected in *Rac1−/−* tissues, but this was mainly confined to cells within the surrounding stromal/fat pad areas. Taken together, these results suggest that the delayed alveolar regression in early involution in *Rac1−/−* glands is linked to increased compensatory cell proliferation within the irreversible phase and not delayed cell death within the reversible phase. Thus, Rac1 has a key role in balancing the rate of cell death and proliferation in involution by limiting the division progeny of progenitors. Without Rac1, alveolar progenitors divide unexpectedly in involution. The newly replaced cells, however, have a limited life span and succumb to death as evidenced by subsequent alveolar regression 4 weeks postweaning involution.

### Loss of Rac1 elicits distinct proliferation responses within the first and second gestation

**Fig 4:**
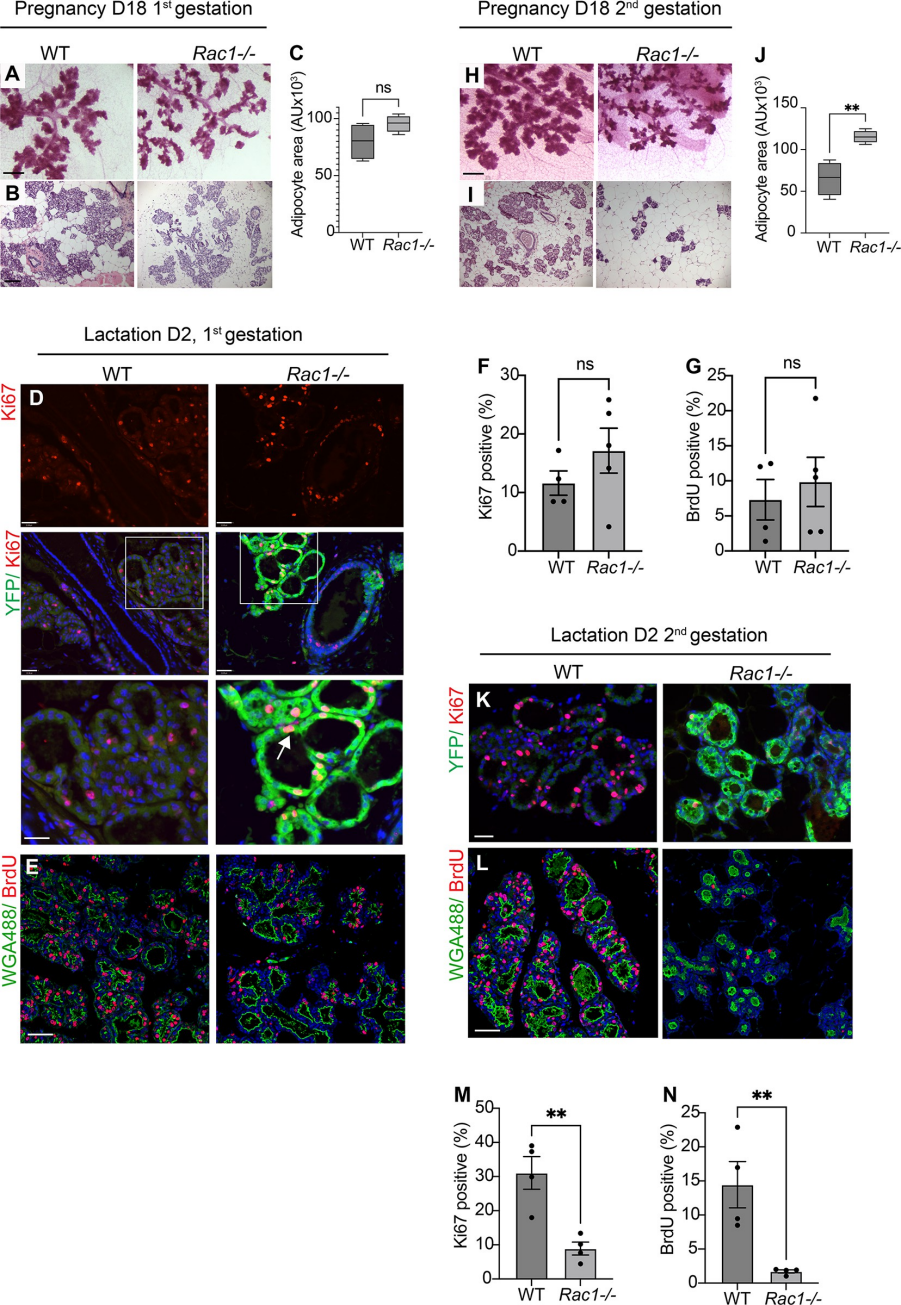
Distinct proliferation in *Rac1−/−* mammary glands within the first and second gestations. (**A**, **B**) Carmine staining of whole-mounted mammary glands (**A**) and HE stain (**B**) from WT and *Rac1−/−* mice at pregnancy day 18 in the first gestation. Bar: 2.8 mm (**A**) and 100 μm (**B**). (**C**) Quantification of adipocyte areas from HE stains show no significant difference. Error bars: +/− SEM of *n =* 4 mice per group. (**D**) Immunofluorescence staining with Ki67 in WT and *Rac1−/−* glands shows no difference in proliferation at lactation day 2 following the first gestation. Green fluorescent protein antibody was used to detect the WAPiCre-driven YFP reporter gene expression and hence Rac1 deletion. Arrows show proliferation in YFP-positive/*Rac1−/−*cells. Bar: 40 μm. (E) BrdU incorporation (red) to detect proliferating cells in WT and *Rac1−/−* mammary glands at lactation day 2, first cycle. WGA-488 (green) was used to demark mammary gland luminal cells. Bar: 40 μm. (**F**, **G**) Quantitative analysis of Ki67 staining (**F**) and BrdU (**G**) at lactation day 2 following the first gestation shows no significant difference between WT and *Rac1−/−* glands. Error bars: +/− SEM of *n =* 4 WT mice and *n* = 5 *Rac1−/−* mice. *P* > 0.05. (**H**, **I**) Carmine staining of whole-mounted mammary glands (**H**) and HE stain (**I**) from WT and *Rac1−/−* mice at pregnancy day 18 in the second gestation. Note the reduced lobular alveolar development. Bar: 2.8 mm (**H**) and 100 μm (**I**). (**J**) Quantification of adipocyte areas from HE stains show increased adipocyte area, thereby reduced alveologenesis in *Rac1−/−* mice. Error bars: +/− SEM of *n* = 4 mice per group. **P ≤ 0.01. (**K**, **L**) Ki67 staining (**K**) and BrdU incorporation (**L**) reveal reduced proliferation in *Rac1−/−* mammary glands at lactation day 2 following the second gestation. Bar: 40 μm. (**M**, **N**) Quantitative analysis of ki67 (**M**) and BrdU (**N**) positive staining in WT and *Rac1−/−* glands at lactation day 2, second gestation. Error bars: +/− SEM of *n* = 4 mice per group. **P ≤ 0.01. The data underlying the graphs shown in this figure can be found in S1 Data.

Rac1 has been linked to cell cycle progression in numerous cultured cells and *in vivo* tissues [19–22]. This is in complete contrast to our findings in vivo in the involuting mammary gland where loss of Rac1 induces proliferation. We thus examined the effects of Rac1 deletion on glandular development and proliferation within the first and second gestation. In the first gestation, histological analysis of mammary glands at pregnancy day 18 revealed slightly smaller alveoli in *Rac1−/−* glands, but the area occupied by adipocytes was not significantly different (Fig 4A–4C). Immunostaining with Ki67 and BrdU incorporation at lactation day 2 in the first cycle revealed no significant difference in proliferation between WT and *Rac1−/−* glands (Fig 4D–4G). Moreover, detection of the *Rosa*:*LSL*:*YFP* reporter gene revealed that recombination and thereby gene deletion was extensive and the proliferation was occurring within YFP-positive/*Rac1−/−* cells and not from WT cells that had escaped recombination (Fig 4D).

In marked contrast to the involuting gland, within the second lactation cycle at day 2, there was a severe block in proliferation of luminal cells with concomitant reduced lobular alveolar development in both late pregnancy and early lactation stages (Figs 4H–4N and S3). Taken together, these data show that Rac1 deletion has no effect on proliferation within the first lactation, heightened proliferation in postlactational involution, and severely defective proliferation within the second lactation. The disparity in proliferation profiles suggests stage-specific cell autonomous and possible nonautonomous regulation by Rac1.

### Increased proliferation in involuting *Rac1−/−* glands is linked to inflammatory signals

**Fig 5:**
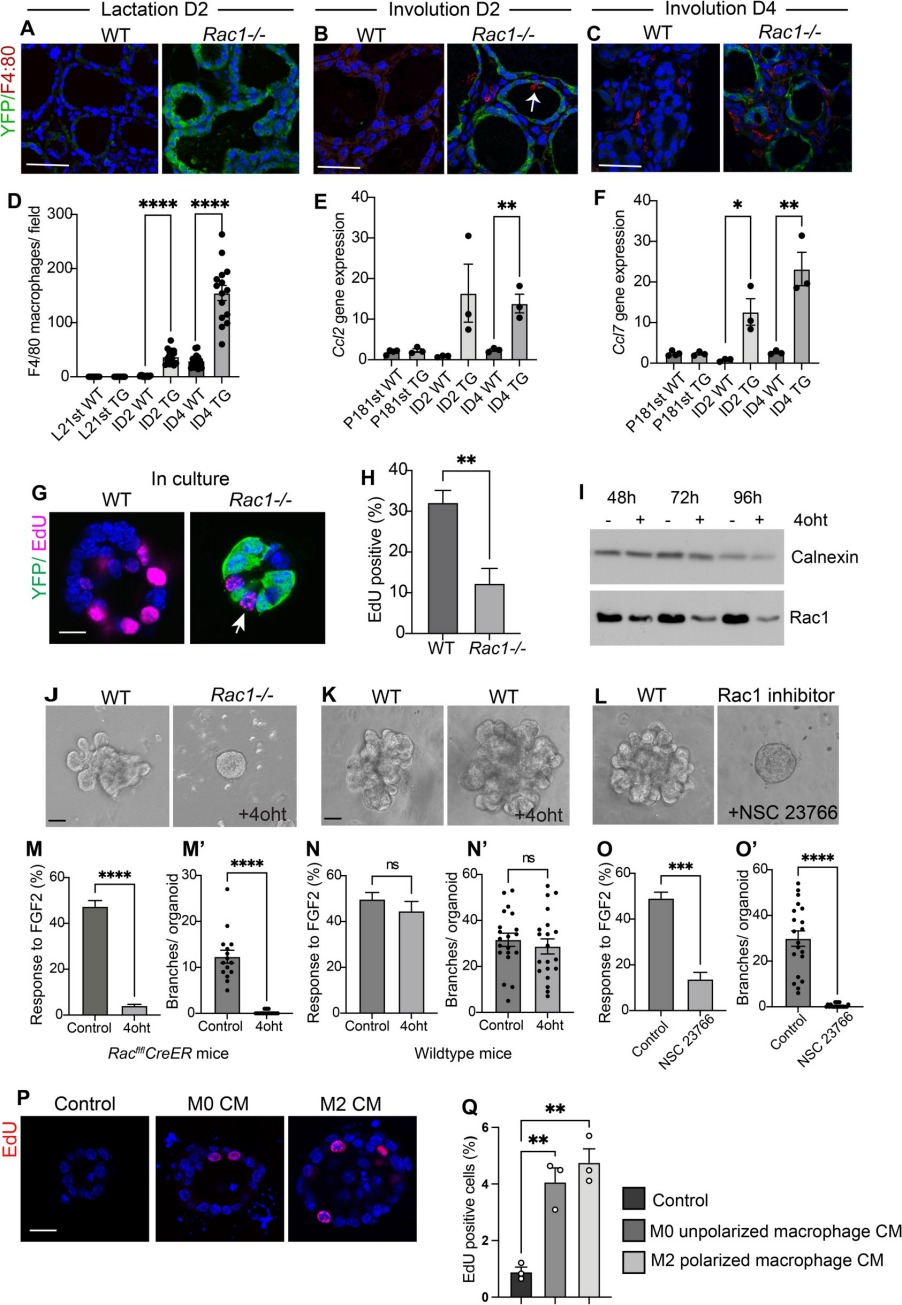
*Rac1−/−* hyperproliferation in involution is linked to inflammation. (**A**-**C**) Immunofluorescence staining with F4/80 antibody (red) to detect macrophages in WT and *Rac1−/−* glands at lactation day 2 and involution days 2 and 4. GFP antibody (green) was used to detect the YFP reporter gene expression. Arrow; macrophage in lumen of *Rac1−/−* alveoli. Bar: 40 μm. (**D**) Quantification of F4/80-positive macrophages shows elevated numbers in *Rac1−/−* transgenics at involution days 2 and 4. No macrophages were detected at the lactation stages. Histogram shows average number of macrophages per field. Error bars: +/− SEM of 15 fields from *n =* 3 mice per group. **** *P* ≤ 0.0001. (**E**, **F**) Quantitative RT-PCR shows elevated inflammatory chemokines CCL2 and CCL7 in *Rac1−/−* glands at involution days 2 and 4 but not in late pregnancy. Error bars: +/− SEM of *n =* 4 mice per group (P18) and *n* = 3 mice per group (Inv D2 and 4). ** *P* ≤ 0.01, * *P* ≤ 0.05. (**G**) EdU incorporation in WT and *Rac1−/−* primary mammary alveolar organoids from *Rac*^*flfl*^*CreER* mid-pregnant mice, cultured on a BM-matrix, show reduced proliferation in the absence of Rac1. Immunofluorescence staining with GFP antibody was used to detect the YFP reporter gene expression (green) and EdU (magenta). Images are confocal sections through the middle of the alveoli. Arrow: proliferation in YFP-negative cells in *Rac1−/−* organoid. Bar: 20 μm. (**H**) Quantitative analysis of EdU incorporation reveals decreased proliferation in *Rac1−/−* organoids. EdU incorporation was counted in YFP-positive cells/*Rac1−/−* only in 4oht-treated organoids. Error bars: +/− SEM of *n =* 3 experiments. **P ≤ 0.01. (**I**) Depletion of Rac1 in primary MEC organoids was confirmed by immunoblotting cell lysates prepared from control or 4oht-treated cultures with a Rac1 antibody. Calnexin antibody was used to show equal loading of protein. (**J**-**L**) Ductal branching in primary culture organoids in response to FGF2 stimulation for 7–8 days. (**J**) Cultures from *Rac*^*flfl*^*CreER* mice show deletion of Rac1 (4oht treatment for 48 h) prevents outgrowth. (**J**) Cultures from WT (CD1 mice) treated with 4oht show outgrowth. (**K**) Rac1 inhibitor (NSC 23766) treatment prevents outgrowth. Bar: 40 μm. (**M**-**O**) Quantitative analysis of organoid numbers responding to FGF2. Error bars: +/− SEM of *n* = 6 coverslips from 2 organoid preps. (**M’**-**O’**) Number of branches per organoid was quantified. Error bars: +/− SEM of *n* = 15–20 organoids. **** *P* ≤ 0.0001, ***P ≤ 0.001, ns = *P* > 0.05. (**P**) EdU incorporation in primary MEC cultures embedded in a BM-matrix and cultured in unconditioned media, or conditioned media (CM) from unpolarised macrophages (M0) or M2 polarised macrophages (M2). (**Q**) Quantitative analysis of EdU incorporation reveals a small increase in proliferation of MECs in response to the macrophage conditioned media. Error bars: +/− SEM of *n* = 3 experiments. **P ≤ 0.01. The data underlying the graphs shown in this figure can be found in S1 Data.

To explain the contrasting effect on cell proliferation within different stages of the mammary gland cycle, we sought to investigate possible cell nonautonomous effects of Rac1. We previously showed heightened inflammatory responses in *Rac1−/−* mammary glands in postlactational involution [9]. Heightened inflammation has been linked to altered epithelial proliferation and pathogenesis within numerous tissues including the gut and skin; we thus sought to investigate whether altered inflammatory responses without Rac1 were linked to the increased proliferation. To test this, we first examined for presence of inflammatory signals within in the gestational stage and in involution. F4/80-positive macrophages and the macrophage recruitment and polarising chemokines CCL2 and CCL7 previously identified in gene expression arrays were used as markers of inflammation [9]. In the first gestation, Rac1 deletion did not induce inflammatory signals (Fig 5A and 5D–5F), and this correlated with no significant difference in proliferation (Fig 4D–4G). At involution day 2, Rac1 deletion heightened inflammatory signals with early macrophage recruitment [9] (Fig 5B, 5D and 5E), and this correlated with elevated proliferation but only within the stromal regions (Fig 3F–3H). At involution day 4, the heightened and sustained inflammation (Fig 5C, 5D and 5F) correlated with increased proliferation in luminal epithelia (Fig 3G–3I, 3L and 3M). This suggests that the inflammatory signals precede luminal cell proliferation and that sustained exposure likely induces the luminal cells to enter the cell cycle.

To remove possible impending signals from inflammatory cells, pure populations of mammary epithelia were isolated from pregnant *Rac1*^*fl/fl*^*CreER* mice and the Rac1 gene was ablated in culture with 4-hydroxytamoxifen (4oht). At this stage, greater than 90% of the cells are alveolar in origin. Loss of Rac1 impaired proliferation in 3D organoids on a basement membrane matrix (Fig 5G–5I). Moreover, ductal cultures from either nulliparous *Rac1*^*fl/fl*^*CreER* mammary glands with 4oht-inducible Rac1 gene deletion or wild-type glands treated with a Rac1 inhibitor failed to branch in response to FGF2 treatment (Fig 5J, 5L, 5M, 5M’, 5O and 5O’). In contrast, treatment of wild-type MECs with 4oht for the same period did not perturb branching morphogenesis over 7 to 8 days (Fig 5K, 5N and 5N’).

To test if morphogens released by macrophages cause proliferation in MECs, primary organoids embedded in a BM-matrix were cultured in conditioned media from M0 or M2 polarised macrophages. EdU incorporation showed a small but significant increase in proliferation in conditioned media compared to control (Fig 5P and 5Q). Together, these data suggest that increased proliferation in involution in *Rac1−/−* glands is in part driven through secondary non-cell-autonomous effects of Rac1 deletion and is connected to heightened and sustained inflammatory responses. These findings reveal that Rac1 mediates a functionally important cross-talk between mammary gland cells and immune phagocytes within their microenvironmental niche.

### Rac1 directs cell death with autophagy but not apoptosis or necrosis

**Fig 6:**
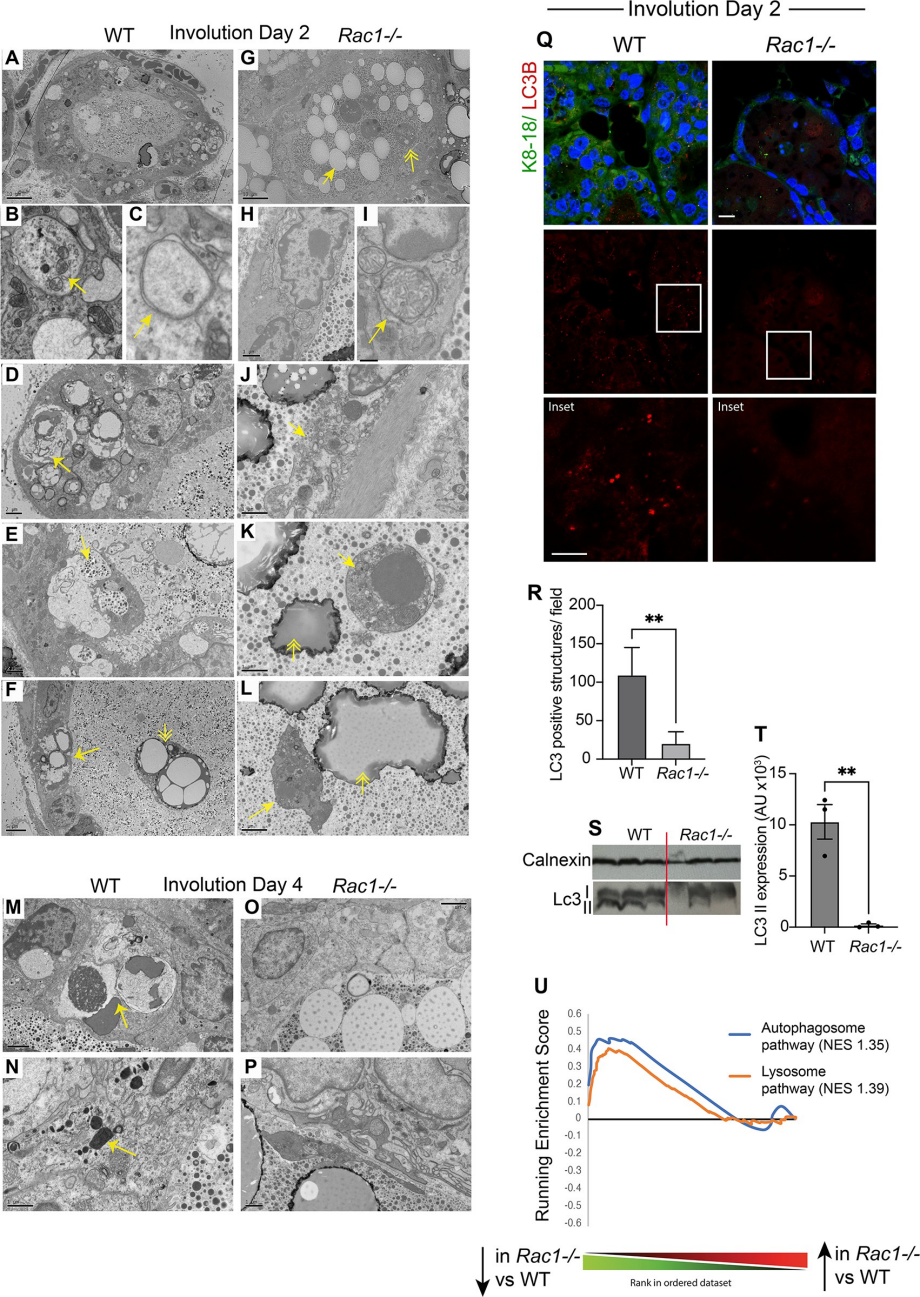
Rac1 mediates cell death with autophagy but not programmed cell death. Electron micrographs of WT (**A**-**F**) and *Rac1−/−* (**G**-**L**) involution day 2 glands. (**A**) WT alveolus showing engulfment activity with numerous phagosome-like structures within the epithelium. Bar: 10 μm. (**B**, **C**) Autophagosomes in WT luminal cells. Note mitochondria in autophagosomes (**B**; arrow) and characteristic double membrane structures (**C**; arrow). (**D**) Autophagosomes and phagosomes containing milk lipids (arrow) in WT cells. Bar: 2 μm. (**E**) Macropinosomes engulfing milk (arrow) in WT cells. Bar: 2 μm. (**F**) Engulfed milk lipid droplets in dying WT luminal cell within the epithelium (arrow) and dead cell shed into the lumen containing engulfed milk lipid droplets (double arrowhead). Bar: 5 μm. (**G**) *Rac1−/−* alveolus showing milk lipid droplets (arrow) and dead cells (double arrowhead) in the lumen but no phagosome-like structures within the epithelium. Bar: 10 μm. (**H**, **I**) No autophagosomes in *Rac1−/−* epithelium. Arrow points to swollen mitochondria. Bar: (**H**) 1 μm, (**I**) 0.4 μm. (**J**) Necrotic (arrow) luminal cell within *Rac1−/−* epithelium. Bar: 1 μm (**K**, **L**) Apoptotic *Rac1−/−* cells shed into the lumen with nuclear pyknosis (**K**; arrow) and membrane blebbing (**L**, arrow). Double arrowheads point to milk lipid droplets in the lumen. Bar: (**K**) 1 μm, (**L**) 2 μm. (**M**-**P**) Involution day 4 WT cells showing autophagosomes, phagosomes (arrow; **M**), and lysosomes (arrow; **N**), but *Rac1−/−* (**O**, **P**) show none. Bar: (**M**, **O**) 2 μm, (**N**, **P**) 1 μm. (**Q**) LC3β antibody was used to detect autophagic structures in involution day 2 tissues. Note: vesicles in WT epithelium but not in *Rac1−/−*. Krt8/18 antibody was used to mark luminal cells. Bar: 10 μM (insert 6 μm). (**R**) Quantitative analysis shows markedly reduced LC3β vesicles in *Rac1−/−* cells. LC3-positive structures were counted per field. Error bars: +/− SEM of *n =* 3 mice. ** *P* ≤ 0.01. (**S**) LC3I and II expression by immunoblot with the LC3β antibody in involution day 2 WT and *Rac1−/−* tissues. Calnexin was used as a loading control. (**T**) Quantification of LC3II band after normalisation to calnexin loading control. Error bars: +/− SEM of *n* = 3 mice. **P ≤ 0.01. (**U**) GSEA demonstrating down-regulation of autophagosome and lysosome genes in *Rac1−/−* glands compared with WT at involution day 2. NES; normalised enrichment score. The data underlying the graphs shown in this figure can be found in S1 and S2 Data files and S1 Table.

As cell death and inflammation are intimately linked, we examined the effects of removing Rac1 on the cell death route in involution. Ultrastructural studies in days 2 and 4, involuting glands revealed numerous vacuolar structures in WT alveolar epithelium but not *Rac1−/−* transgenics (Figs 6A, 6G, 6M, 6O and S4). Further analysis revealed autophagosomes and lysosomes in the WT alveolar epithelium, suggestive of cell death with autophagy (Figs 6A–6C, 6M, 6N and S4). Moreover, we detected numerous phagosomes with engulfed milk proteins, milk lipid droplets, and dead cells (Fig 6D–6F), which supports our previous findings showing engulfment through phagocytic cups and macropinosomes in WT cells [9]. In contrast, *Rac1−/−* alveoli were completely void of both autophagosomes and phagosomes; instead, we detected either live cells in the epithelium or dead cells shed into the lumen with late apoptotic morphology, and some necrotic cells with ruptured membranes and organelles released extracellularly (Fig 6G–6L, 6O and 6P). We further confirmed autophagy in WT glands by immunostaining for the essential autophagy-related LC3β protein, which appeared punctate and therefore indicative of translocation to the autophagosome membrane. In contrast, LC3β staining was diffuse in *Rac1−/−* transgenics confirming the absence of autophagosomes (Fig 6Q and 6R). Moreover, immunoblotting with the LC3β antibody showed reduced LC3II in *Rac1−/−* glands (Fig 6S and 6T). Consistent with the ultrastructural studies, gene array analysis revealed down-regulation of genes associated with autophagosome and lysosomal pathways (Fig 6U). Whole groups of lysosomal hydrolases, including proteases, glycosidases, sulfatases, DNases, and lipases, were down-regulated in *Rac1−/−* transgenics (S1 Table). Taken together, these data show that Rac1 is required to induce cell death with autophagy but without Rac1 cells can still die via apoptosis and necrosis. The presence of necrotic cells likely contributed to the heightened inflammatory responses in *Rac1−/−* glands.

### Cell death is accelerated without Rac1

**Fig 7:**
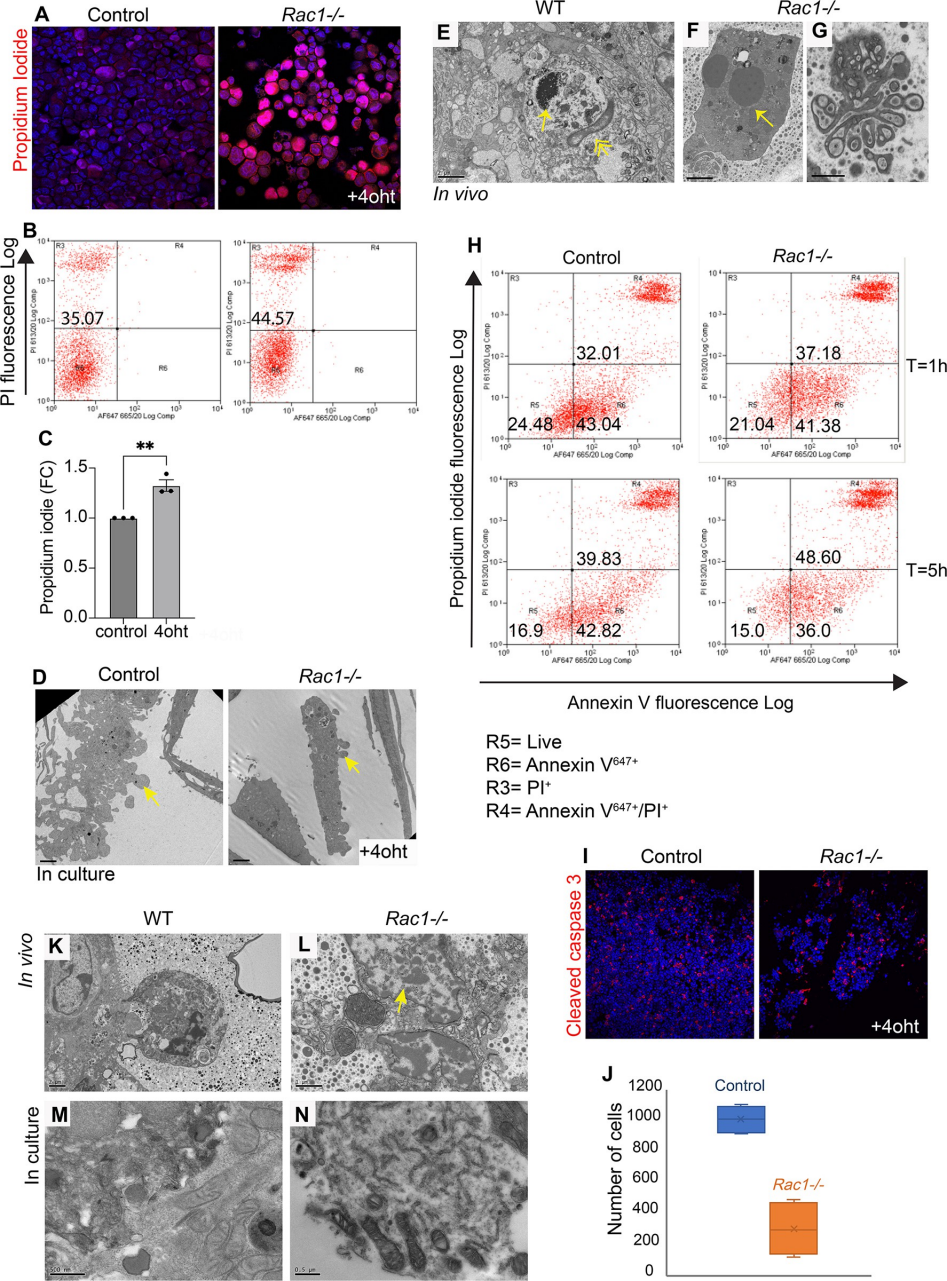
Cell death proceeds faster without Rac1. (**A**-**C**) Increased PI uptake in *Rac1−/−* cells induced to die through anoikis in culture; (**A**) fluorescent image and (**B**) FACS analysis (**C**) Quantification of FACS. FC (fold change). Error bars: +/−SEM of *n =* 3 preps. **P ≤ 0.01. (**D**) Apoptotic blebs in WT and *Rac1−/−* dead cells in culture detected using EM. Arrows: membrane blebs. Bar: 2 μm. (**E**-**G**) EM images of (**E**) WT and (**F**, **G**) *Rac1−/−* tissues in vivo show apoptotic cells with nuclear pyknosis (**E**, **F**; arrows) and membrane blebbing (**G**). Double arrowhead: Apoptotic cells are engulfed by the alveolar epithelium in WT glands (**E**). Bar: (**E**, **F**) 2 μm, (**G**) 1 μm. (**H**) Annexin-V^647+^ and PI^+^ colabelled WT and *Rac1−/−* cells quantified by flow sorting following 1 h and 5 h in suspension show more cells proceed to late-stage apoptosis/necrosis without Rac1. (**I**) WT and *Rac1−/−* cells induced to die through anoikis for 8 h show reduced numbers of viable cells without Rac1. Cleaved caspase-3 was used to stain apoptotic cells. (**J**) Quantification of (**I**) showing total number of WT and *Rac1−/−* cells. Error bars: +/− SEM of *n* = 4. (**K**-**N**) EM images of WT and *Rac1−/−* in vivo tissues (**K**, **L**) and primary cultures (**M**, **N**) show cell necrosis without nuclear pyknosis and organelle release in *Rac1−/−* cells (**L**, **N**). In contrast, dying WT cells (**K**, **M**) have an intact cell membrane. Arrow: Nucleus released from necrotic cell without condensation suggests direct necrosis. Bar: (**K**) 2 μm, (**L**) 1 μm, (**M**, **N**) 0.5 μm. The data underlying the graphs shown in this figure can be found in S1 Data.

We next investigated whether Rac1 affects the rate at which cells transit through death and whether cells die directly through primary or secondary necrosis. To test this, primary cultures of WT and *Rac1−/−* cells were induced to undergo anoikis, a detachment-induced cell death. We chose this method of cell death for three reasons; first, to prevent dying cells from removal by neighbouring nonprofessional phagocytosis, as single cells suspended in media are spatially out of reach for phagocytic removal; second, to trigger an innate programmed cell death rather than chemical-induced; and third, anoikis likely occurs in *Rac1−/−* glands in vivo as we previously showed loss of Rac1 perturbs cell-ECM adhesion with increased shedding of cells [9]. Dying cells incorporated higher levels of propidium iodide (PI) in the absence of Rac1 compared to WT controls, suggesting cell death by necrosis or late-stage apoptosis (Fig 7A–7C). To establish the proximal cell death route, we first examined for hallmarks of apoptosis as this process accompanies a series of well-defined biological steps. Both WT and *Rac1−/−* cell corpses displayed intact membranes with nuclear condensation, late-stage membrane blebbing, body fragmentation, and stained positive for cleaved caspase 3 indicative of an apoptotic cell death (Figs 3B, 3E and 7D–7G). Early-stage apoptosis is characterised by phosphatidylserine exposure to the outer membrane leaflet, and Annexin V is commonly used to detect this motif. Colabelling with Annexin V and PI in cells suspended for 1 h and 5 h revealed that approximately the same number of cells enter apoptosis with and without Rac1 (Annexin V only); however, by 5 h, significantly more *Rac1−/−* cells proceed to late-stage apoptosis/necrosis (Annexin V/ PI) with a concomitant reduction in numbers in early-stage apoptosis (Annexin V only; Fig 7H). Moreover, the numbers of viable cells declined following an 8-h suspension in the absence of Rac1 indicating that cells had proceeded through death and disintegrated within this time frame compared to WT controls (Fig 7I and 7J).

As Annexin V can also bind necrotic cells with ruptured membranes, some of the cells in the AnnexinV/PI fraction may be a result of primary necrosis. To address whether necrotic cells occurred secondary to apoptosis as a result of defective phagocytosis or whether Rac1 loss triggered necrosis, we analysed the nuclei of necrotic cell corpses by electron microscopy. In *Rac1−/−* glands, the nuclei of ruptured cells were not condensed, suggesting they had not entered the apoptotic pathway first, but rather died through primary necrosis (Fig 7L). Organelle spillage and cell necrosis were also detected in *Rac1−/−* primary cultures (Fig 7N). In contrast, WT dead cells had intact membranes with nuclear condensation (Fig 7K and 7M). These studies reveal that Rac1 slows down the process of programmed cell death. Without Rac1, cells die either through primary necrosis or through apoptosis, but death proceeds more rapidly than in WT epithelia. Taken together, loss of Rac1 increases cell turnover rates in the involuting mammary gland through both increased progenitor proliferation and accelerated cell death.

### Lactation fails to resume upon pup resuckling in involuting *Rac1−/−* glands

**Fig 8:**
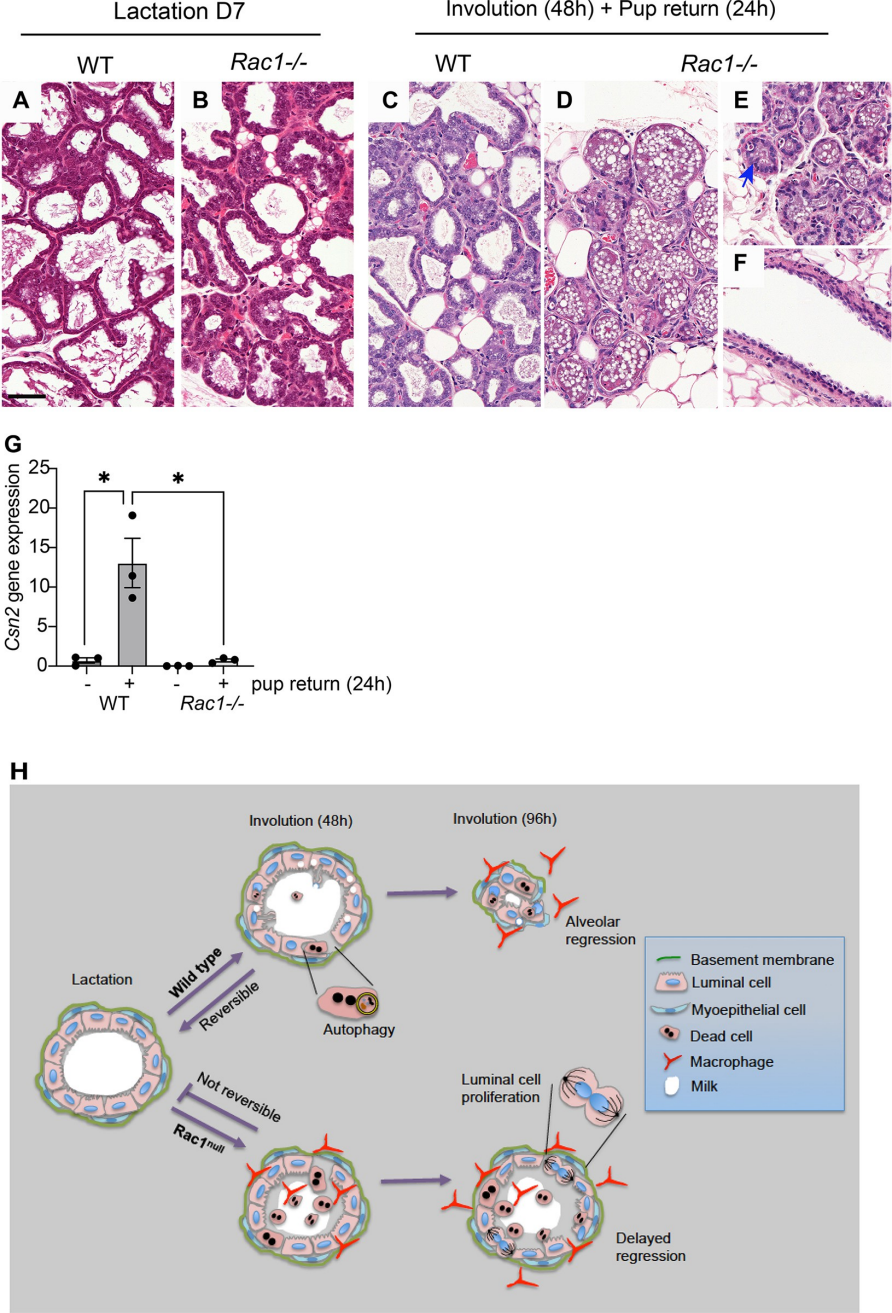
Mammary gland reversibility fails in *Rac1−/−* involuting glands. (**A**-**F**) HE stain of WT and *Rac1−/−* glands at lactation day 7 (**A**, **B**) and involuted for 48 h and then resuckled for 24 h (**C**-**F**). WT glands (**C**) resume a lactation morphology while *Rac1−/−* (**D**-**F**) show an involution phenotype. (**E**, **F**) show suckled areas in *Rac1−/−* glands. Bar: 100 μm. (**G**) Quantitative RT-PCR shows increased *casein 2* gene expression in resuckled WT glands, but this is reduced by 18-fold in *Rac1−/−* glands. Error bars: +/−SEM of *n =* 3 mice. (**H**) Summary diagram of Rac1 regulation of mammary gland reversibility in involution. Rac1 permits cell death with autophagy, which enables reversibility within the first 48 h if suckling recommences. Without Rac1, early inflammation, a lack of autophagy and concomitant alternative programmed cell death, prevents redifferentiation of cells. At 96 h, Rac1 mediates remodelling of mammary gland tissue with alveolar shrinkage. Without Rac1, heightened inflammatory signals stimulate luminal cell proliferation and the alveoli remain distended. The data underlying the graphs shown in this figure can be found in S1 Data.

To determine the functional consequences of the phenotypic defects in *Rac1−/−* glands, we investigated the reversible phase of the involution process. Nursing WT and *Rac1−/−* dams were separated from the pups for 48 h to stimulate the first phase of involution and then reunited for a 24-h period. Resuckling in the WT mammary glands recommenced lactation as detected by an approximate 18-fold increase in *casein 2* gene expression, and the alveolar epithelium resumed a lactation morphology (Fig 8A, 8C and 8G). In contrast, the *Rac1−/−* glands failed to lactate, and the involution morphology persisted with dead cell shedding into the lumen (Fig 8D–8F and 8G). While a small increase in milk protein gene expression was detected in resuckled *Rac1−/−* glands compared with the involuting *Rac1−/−*, the *casein* gene expression was 18-fold less than the WT fed gland. This compromise in milk protein expression is significantly greater than the 2-fold decrease detected in the first lactation cycle where *Rac1−/−* dams are still able to support pups [9]. Together, these data indicate that Rac1 is crucial for mammary gland reversibility in phase I of the involution process upon resuckling. Without Rac1, alveolar cells fail to redifferentiate and lose the ability to recommence lactation (Fig 8H).

## Discussion

Our study reveals that Rac1 acts as a central nexus in controlling the balance of cell death and proliferation within the mammary gland and is critical for mammary gland reversibility in early involution. The mammary gland removes approximately 90% of its tissue weight in postlactational involution with mass destruction of the milk-secreting alveolar units. This extensive regression can only be accomplished if the balance tips towards cell death with reduced proliferation. We have discovered that Rac1 is central to maintaining the suppression of proliferation in involution. Removal of Rac1 induces extensive proliferation within the involuting gland within the irreversible phase. We show the initial delay in alveolar regression and concomitant fat pad repopulation is not due to delayed cell death but rather a lack of milk engulfment [9] accompanied by compensatory cell proliferation within alveoli. However, the newly replaced cells in alveoli have a short life span, as the alveoli regressed by 4 weeks postweaning. A recent study reported that engulfed milk lipids are recycled to expand adipocytes [23]. Thus, the delay in adipocyte repopulation in *Rac1−/−* tissues might not be solely due to space constraints but also availability of engulfed lipids for recycling.

A previous study also reported a delay in *Rac1−/−* alveolar regression but attributed the effects to delayed cell death [24]. In contrast, our data show that cell death is not blocked in *Rac1−/−* alveoli in early involution as we have detected numerous cell corpses using both light and electron microscopy.

The involution process is accompanied by inflammatory cell influx to remove residual dead corpses by phagocytosis, increased matrix metalloproteinase activity, and extracellular matrix remodelling with subsequent release of various morphogens [13,25]. It is well established that proinflammatory signals invoke stem cell proliferation in several disease models including psoriasis and various cancers. Accumulating evidence shows that the involution microenvironment supports breast cancer growth in tumour mouse models and promotes postpartum breast cancer in women [25–29]. Of interest is a study showing sustained up-regulation of the chemokine CCL2 increased cancer susceptibility in a transgenic mouse model [30]. Loss of Rac1 increases proinflammatory signals in the mammary gland including the chemokines CCL2 and CCL7. We have made the important discovery that one mechanism by which the postpartum involuting mammary gland protects itself from inflammation-induced proliferation is through the Rac1 GTPase. Interestingly, in the skin, loss of Rac1 also causes stem cell release through activation of c-myc, although this study did not investigate inflammatory responses [31]. Whether Rac1 promotes or suppresses cell proliferation appears to be dependent on the microenvironmental context. Rac1 is linked to stem cell renewal and cell cycle progression in mammary epithelia and in numerous other models [17,19–22]. We have now shown that Rac1 genetic deletion perturbs proliferation in purified epithelial organoid cultures void of impending inflammatory cells. In contrast, organoids exposed to inflammatory signals proliferate. This suggests both cell autonomous and nonautonomous regulation of proliferation by Rac1 depending on the environmental context. In addition to heightened inflammatory signals, stagnant milk in *Rac1−/−* mammary gland structures linked to the defective engulfment might cause stretch-induced proliferation. Indeed, ductal and alveolar bloating is severe in *Rac1−/−* glands because of defective clearance by MEC phagocytes [9]. Studies in the *Drosophila* wing disc show dying cells promote growth in their neighbours through the Wnt family member Wingless and Dpp proteins [32,33]. It would be interesting to identify whether similar autonomous regulation also occurs in the mammary gland.

Upstream of Rac1, β1-integrin has been linked to stem cell renewal, cell cycle progression, and lactational differentiation in mammary epithelia [16,18,19,34,35]. Here, we demonstrate that in involution, Rac1 controls alveolar regression independently of β1-integrin. This suggests distinct upstream wiring allows Rac1 to perform multifaceted roles within the mammary gland. The Rac guanine nucleotide exchange factors (GEFs) ELMO and Dock180 also show delayed alveolar regression in involution, but the receptor that activates these GEFs remains to be identified [24].

Our data show that alveolar epithelial cells die through distinct mechanisms with and without Rac1. Multiple cell death mechanisms have been reported in mammary gland involution, including autophagy and lysosomal leakiness linked to milk fat engulfment [4,5,36]. Executioner caspases 3, 6 are detected in cell corpses in the first 48 h suggesting activation of these pathways, although once ablated cell death can also proceed independently of these caspases [4]. We discovered that in the absence of Rac1, cell death with autophagy is impaired; milk phagocytosis is impaired [9] with a concomitant lack of detectable lysosomes by EM and down-regulation of several lysosomal genes. Despite these deficiencies, *Rac1−/−* cells still die through apoptosis and necrosis, and there is no delay in cell death. These alternate routes ensure a protective redundancy that enables cell death to proceed. Of interest is that in the autophagy defective *Beclin 1−/−* mammary glands, Rac1 activation is perturbed, which suggests a regulatory feedback loop [5].

We have discovered that, in addition to increased proliferation, cell transit through the death process is accelerated without Rac1, thereby Rac1 critically functions to limit cell turnover rates in involution. This suggests a mechanism by which existing cells resist cell death to allow reversibility. One such mechanism is autophagy, which might act as a survival mechanism in early involution instead of cell death. We have identified this as an important self-regulatory mechanism that enables mammary gland reversibility in involution. Despite the presence of live alveolar cells within *Rac1−/−* alveoli in the first 48 h, the dedifferentiated cells cannot lactate upon resuckling. The lactation defect is long term, as we have previously demonstrated severely defective future lactations in *Rac1−/−* mammary glands [9]. Future studies will focus on how perturbing Rac1 alters the luminal stem/progenitor niche leading to defective alveolar lineages and long-term tissue malfunction in successive gestations.

## Methods

### Ethics statement

Experiments with mice were approved by the University of Sheffield AWERB (Animal Welfare and Ethics Review Board) and conducted under the Home Office Licences PPL 70/8351 and PPL 1836785 awarded to NA. Mice were housed and maintained according to the UK Home Office guidelines for animal research.

### Mice

The *Rac1*^*fl/fl*^: *YFP;WAPiCre*^*Tg/•*^ and *Rac1*^*fl/fl*^*;CreER* mice were as previously described [15]. For the in vivo analysis, the WAPiCre promoter, which is activated mid-late pregnancy was used for *Rac1*^*fl/fl*^ gene deletion specifically in luminal MECs. *Rosa*:*LSL*:*YFP* reporter gene was used to detect Cre-induced recombination of flox alleles. *Rac1*^*fl/fl*:^ *YFP* littermates that lacked the *Cre* gene were used as WT controls. The genotypes of offspring were determined by PCR amplification of ear DNA as in [15]. *Rac1*^*fl/fl*^:*YFP;CreER* mice were used for inducible deletion of the Rac1 gene in primary cultures. Female mice were mated between 8 and 12 weeks of age. *β1-integrin*^*fl/fl*^:*YFP*:*WAPiCre* mice were generated by crossing *β1-integrin*^*flfl*^ mice; JAX #004605 [37] with *WAPiCre*:*YFP* mice previously described [9]. For involution studies, dams were allowed to nurse litters (normalised to 6 to 8 pups) for 7 to 10 days, and then pups were weaned to initiate involution. In the involution rescue experiments, breeding trios were set up with a male, an experimental female, and a WT surrogate female. Impregnated females were separated from the male, allowed to litter and nurse offspring jointly as above. Experimental dams were separated for 48 h to involute, while the surrogates continued feeding the pups. Litters were subsequently reunited with the experimental female for a period of 24 h prior to gland harvesting. Around 3 to 5 mice per group were analysed for each developmental stage. For some experiments, mice were injected with BrdU 100 mg/kg for 2 h before harvesting.

### Histology

Mammary tissue was formalin fixed (4% v/v), paraffin embedded before sectioning (5 μm), and subjected to standard haematoxylin–eosin (HE) staining. Histology was imaged using 3D Histotec Pannoramic 250 slide scanner and Aperio ImageScope version 12.1 software. Adipocytes were quantified using Fiji/ImageJ adiposoft plug-in. Five microscopy fields on a 10× lens were quantified.

Whole mount analysis was performed by spreading inguinal mammary glands on polysine slides and stained with carmine alum as previously described [34]. Glands were imaged using a Nikon SMZ18 stereoscope.

### TEM

Involuting mammary tissues were fixed with 4% formaldehyde + 2.5% glutaraldehyde in 0.1 M HEPES buffer (pH 7.2) for 1 h. Postfixed with 1% osmium tetroxide + 1.5% potassium ferrocynaide in 0.1 M cacodylate buffer (pH 7.2) for 1 h, in 1% thyocarbohydrazide in water for 20 min, in 2% osmium tetroxide in water for 30 min, followed by 1% uranyl acetate in water for overnight. The next day tissues were stained with Walton lead aspartate for 1 h at 60°C degree, dehydrated in ethanol series infiltrated with TAAB 812 hard grade resin, and polymerised for 24 h at 60°C degree. TEM sections were cut with Reichert Ultracut ultramicrotome and observed with FEI Tecnai 12 Biotwin microscope at 100 kV accelerating voltage. Images were taken with Gatan Orius SC1000 CCD camera.

### RNA isolation and cDNA synthesis

RNA was isolated from the fourth inguinal mammary gland using Trifast reagent (Peqlab) according to manufacturer’s instructions. Approximately 2 μg of RNA was used to prepare cDNA using the high-capacity RNA to cDNA kit (Invitrogen) according to manufacturer’s instructions.

### Affymetrix gene array

Gene arrays were conducted previously [9]. Data accession: E-MTAB-5019 (Array Express) or GSE85188 (GEO). Gene lists were analysed using DAVID, Panther, and GSEA web accessible programs.

### Quantitative RT-PCR

Quantitative RT-PCR was performed on the Applied Biosystems 7900HT using the following Taqman probes (Applied Biosciences; Life Technologies): Krt18 (Mm01601704_g1), MAPKI (Mm00442479_m1) as housekeeping genes, CCL2 (Mm00441242_m1), CCL7 (Mm00443113_m1), Csn2 (Mm04207885_m1). Thermal cycling conditions were as follows: UNG start at 50°C (2 min), 95°C (10 min), and then 40 cycles of 95°C (15 s) followed by annealing at 60°C (1 min).

### Primary cell culture and gene deletion

Primary MECs were harvested from either 15.5 to 17.5 day pregnant mice for alveolar cultures or 12-week-old virgin mice for ductal cultures and cultured as described in [38].

Cells were plated onto Collagen 1 for monolayer cultures or BM-matrix (Matrigel; Corning B.V. Life Sciences, NBD Biosciences) to form acini and cultured in growth media (Ham’s F12 medium (Sigma) containing 5 μg/ml insulin, 1 μg/ml hydrocortisone (Sigma), 3 ng/ml epidermal growth factor (EGF), 10% foetal calf serum (Biowittaker), 50 U/ml Penicillin/Streptomycin, 0.25 μg/ml fungizone, and 50 μg/ml gentamycin). For ductal branching, cells were cultured in branching medium (DMEM-F12 medium (Lonza) containing insulin-transferrin-selenium G supplement (100×), 2 nM hb-fibroblast growth factor (Sigma cat: F0291) and 50 U/ml Penicillin/Streptomycin) for 7 to 8 days. For some experiments, ductal organoids from wild-type CD1 mice were treated with 50 μM NSC 23766 Rac1 inhibitor. For inducible Rac1 gene ablation, MEC cultures were prepared from *Rac1*^*fl/fl*^*;CreER* mice and treated with 100 nM 4oht for 48 h (added at time of plating). For proliferation assays, 10 μM EdU was added for 2 h to either day 2 or day 3 cultures and developed using the Click-IT EdU Alexa Fluor imaging kit (Life Technologies).

### Macrophage-conditioned media

Murine RAW 264.7 cells were cultured in DMEM (Sigma) supplemented with 1% (v/v) penicillin–streptomycin and 10% foetal bovine serum to 80% confluency. M2 polarisation was induced by adding 20 ng/ml of recombinant mouse IL4 (Bio-Techne) and recombinant mouse IL13 (Bio-Techne) for 24 h. Macrophages were left untreated for M0. Polarisation media was washed off and replaced with serum-free medium to generate conditioned media for 24 h, after which it was collected and filtered through a 0.22-μM pore filter to remove cell debris. Approximately 200 μg/ml Insulin-Transferrin-Selenium (Gibco) and 1 μg/ml hydrocortisone (Sigma) were added to the media.

Primary mammary cells embedded in a BM-matrix were cultured with either unconditioned media or conditioned media from M0 or M2 macrophages. Organoids were treated with 10 μM EdU for 2 h prior to processing.

### Anoikis assays and FACS

Apoptotic MECs (−/+4oht) were prepared in suspension culture for either 1 h or 5 h, in serum-free media (DMEM-F12 containing 2.5 μg/ml insulin). Cells were labelled with PI and/or Alexa Flour 647-Annexin V (Biolegend) according to manufacturer’s instructions and analysed by flow cytometry or cytospun onto polysine slides, fixed and counterstained with Hoechst for micrographs. Some cytospun suspension cultures were stained with cleaved caspase-3 antibody.

### Immunostaining

Expression and distribution of various proteins were visualised by indirect immunofluorescence. Cells were fixed for 10 min in PBS/4% (w/v) paraformaldehyde and permeabilised for 7 min using PBS/0.2% (v/v) Triton X100. Nonspecific sites were blocked with PBS/10% goat serum (1 h, RT) prior to incubation with antibodies diluted in PBS/5% goat serum (1 h, RT, each). EdU was detected using the Click-iT EdU Alexa Fluor 647 imaging kit (Life Technologies), and nuclei were stained using 4 μg/ml Hoechst 33258 (Sigma) for 5 min at RT. Cells were washed in PBS before mounting in prolong gold antifade (Molecular Probes). Images were collected on a Nikon A1 confocal or a Leica TCS SP5 AOBS inverted confocal as previously described [15]. Nonbiased cell counts were performed by concealing the identity of each slide.

Immunostaining of mammary tissue was performed on paraffin-embedded tissue (5 μm) or cryosections (10 μm) as previously described [9]. Wheat germ agglutinin (WGA)-488, or -647 (Invitrogen, #W11261, #W32466), was used for staining the epithelium and imaged using confocal microscopy. Primary antibodies used for immunofluorescence are indicated in S2 Table. Secondary antibodies were conjugated to Cy2, Alexa-488, Rhodamine-RX, Cy5, Alexa-647 (Jackson Immunoresearch). Nonbiased image analysis was performed in 5 to 10 microscopy fields per mouse.

### Protein analysis

Proteins were extracted as in [16]. Equal amounts of proteins were used and equivalent loading assessed by referral to controls, such as Calnexin (Bioquote SPA-860). Primary antibodies used for immunoblotting are indicated in S2 Table. ImageJ was used to quantify bands.

## Supporting information

S1 Fig S1 Fig related to Fig 1. Failed alveolar regression in involuting *Rac1−/−* mammary tissues. (**A**) Representative HE stains of *n =* 3 mice showing delay in alveolar regression and adipocyte repopulation in *Rac1−/−* tissues at involution day 4. Earclip numbers are indicated on the micrographs. Bar: 80 μm.
(PDF)

S2 Fig S2 Fig related to Fig 3. Cell death and proliferation in involuting *Rac1−/−* mammary glands. (**A**) HE stain showing numerous dead cells (arrow) in alveolar lumens of *Rac1−/−* transgenic glands at involution days 1, 2, and 4. Bar: 50 μm. (**B**) Cleaved caspase-3 staining in involution day 2 mammary glands show increased cell corpses in *Rac1−/−* compared to WT. Bar: 200 μm. (**C)** Immunofluorescence staining with Ki67 antibody reveals heightened proliferation at involution day 4 in *Rac1−/−* tissues; *n* = 3 mice shown. SMA was used to stain myoepithelial cells and thereby demark alveolar boundaries. Bar: 200 μm. Micrographs in (**B**, **C**) were taken on a 20× objective lens to show wider areas.
(PDF)

S3 Fig S3 Fig related to Fig 4. Reduced alveologenesis in the second lactation. (**A**, **B**) Carmine staining of whole-mounted mammary glands from WT and *Rac1−/−* mice at lactation day 1 (**A**) and 2 (**B**) following the second pregnancy reveal reduced alveologenesis. Bar: 2.8 mm (insert 0.3 mm).
(PDF)

S4 Fig S4 Fig related to Fig 6. Loss of autophagosomes, phagosomes, and lysosomes in *Rac1−/−* glands. (**A**, **B**) Electron microscopy micrograph showing multiple alveoli from WT (**A**) and *Rac1−/−* (**B**) involution day 2 mammary glands. Note: Numerous autophagosome/phagosome-like structures are evident in WT alveolar epithelia (arrow; **A**) but not in *Rac1−/−* alveoli (arrow; **B**). Instead, *Rac1−/−* alveolar lumens are full of dead cells (arrowheads) and milk lipids and proteins (double arrowhead). Bar: 20 μm. (**C**, **D**) Electron microscopy micrograph showing alveoli from WT (**A**) and *Rac1−/−* (**B**) involution day 4 mammary glands. Note: the collapsed alveoli in WT (arrow; **C**) and multiple lysosomes in cells (arrowheads; **C**). In contrast, *Rac1−/−* alveoli is distended with a lack of lysosomes in cells (arrow; **D**). Bar: 5 μm.
(PDF)

S1 Table S1 Table related to Fig 6. List of lysosomal genes down-regulated in *Rac1−/−* glands. Microarray gene expression data from WT and *Rac1−/−* mammary glands, showing list of lysosomal genes down-regulated in *transgenics*, *n* = 3 mice were used per condition.
(PDF)

S2 Table Primary antibody information. (PDF)

S1 Data Raw data accompanying quantitative analysis in Figs 1–8. (XLSX)

S2 Data Affymetrix gene array data underlying the graph in Fig 6U. (XLSX)

S1 Raw Images Related to Figs 5 and 7. Raw images of western blots.
(PDF)

## Abbreviations

EGF: epidermal growth factor
GEF: guanine nucleotide exchange factor
HE: haematoxylin–eosin
MEC: mammary epithelial cell
PI: propidium iodide
WGA: wheat germ agglutinin
WT: wildtype
4oht: 4-hydroxytamoxifen
