# Supplementary figures and images for: Rac1 controls cell turnover and reversibility of the involution process in postpartum mammary glands
Source: PLoS Biol. 2023 Jan 19;21(1):e3001583. doi: 10.1371/journal.pbio.3001583 (PMC9851507; doi:10.1371/journal.pbio.3001583)

Involution day 4

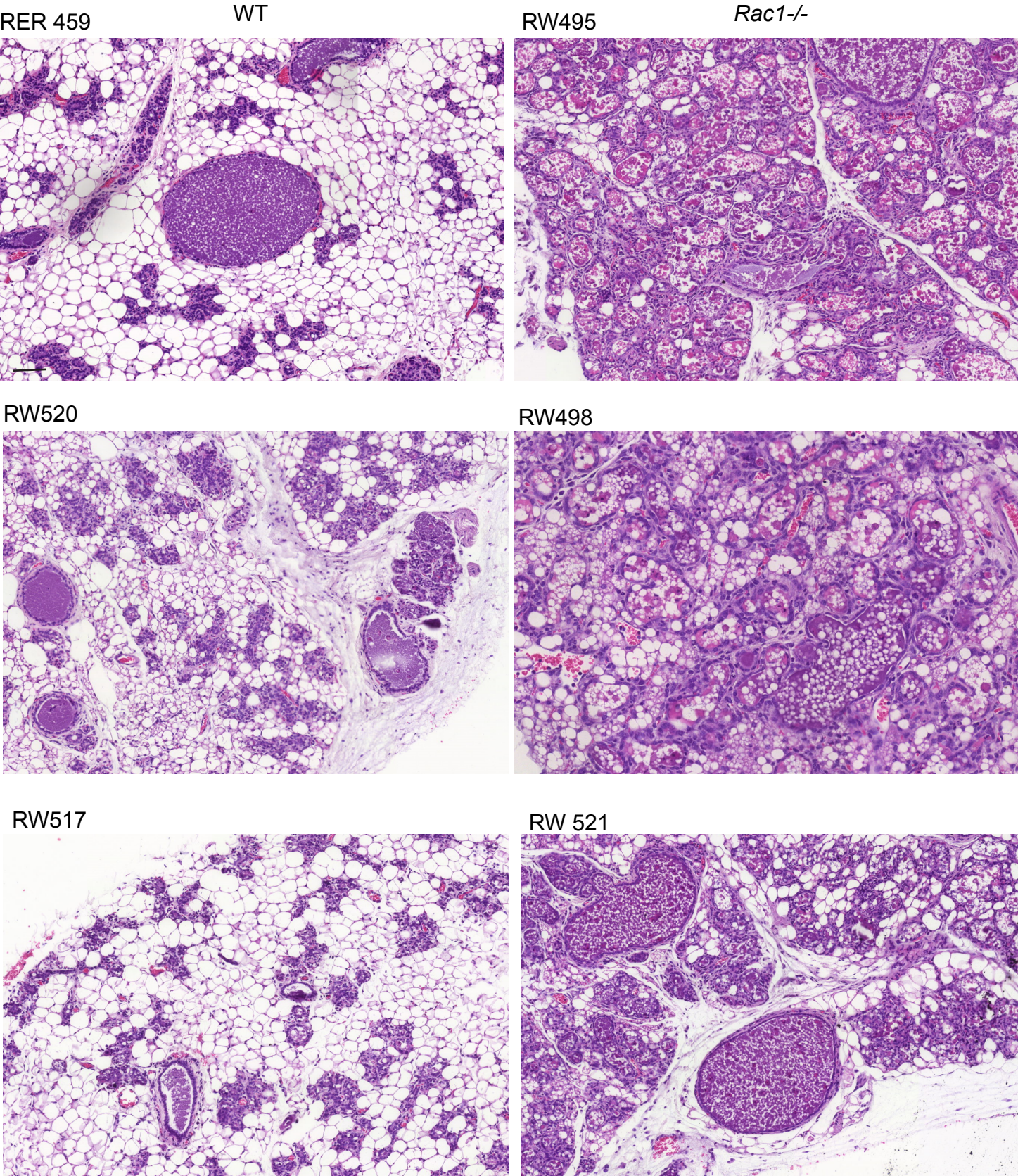

S1 Fig

Supplement: S1 Fig — Failed alveolar regression in involuting Rac1−/− mammary tissues. (A) Representative HE stains of n = 3 mice showing delay in alveolar regression and adipocyte repopulation in Rac1−/− tissues at involution day 4. Earclip numbers are indicated on the micrographs. Bar: 80 μm. (PDF) [file pbio.3001583.s001.pdf]

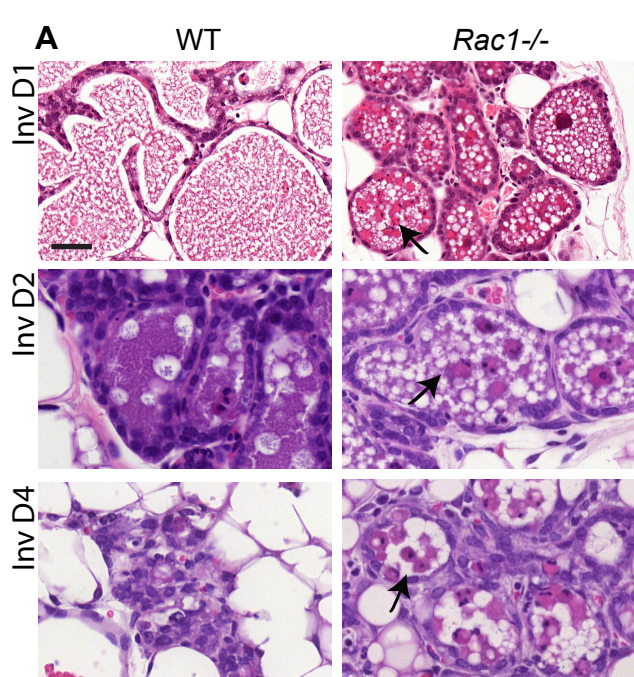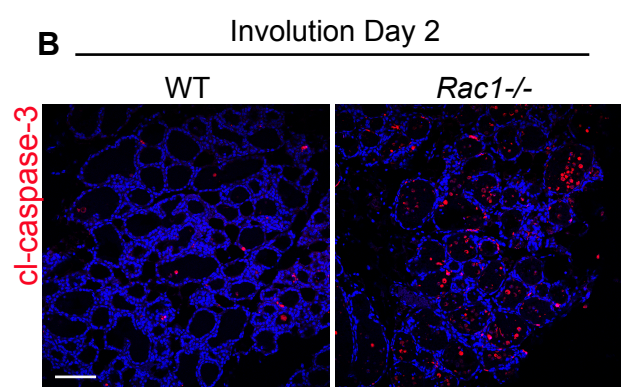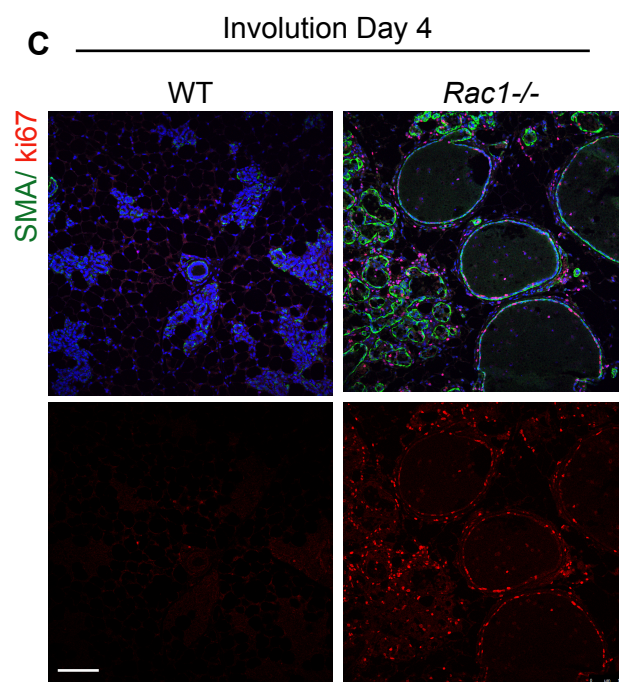

S2 Fig

Supplement: S2 Fig — Cell death and proliferation in involuting Rac1−/− mammary glands. (A) HE stain showing numerous dead cells (arrow) in alveolar lumens of Rac1−/− transgenic glands at involution days 1, 2, and 4. Bar: 50 μm. (B) Cleaved caspase-3 staining in involution day 2 mammary glands show increased cell corpses in Rac1−/− compared to WT. Bar: 200 μm. (C) Immunofluorescence staining with Ki67 antibody reveals heightened proliferation at involution day 4 in Rac1−/− tissues; n = 3 mice shown. SMA was used to stain myoepithelial cells and thereby demark alveolar boundaries. Bar: 200 μm. Micrographs in (B, C) were taken on a 20× objective lens to show wider areas. (PDF) [file pbio.3001583.s002.pdf]

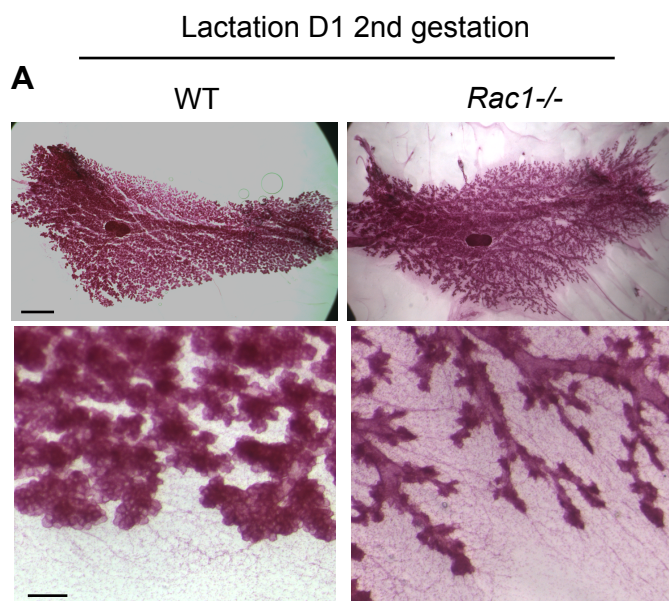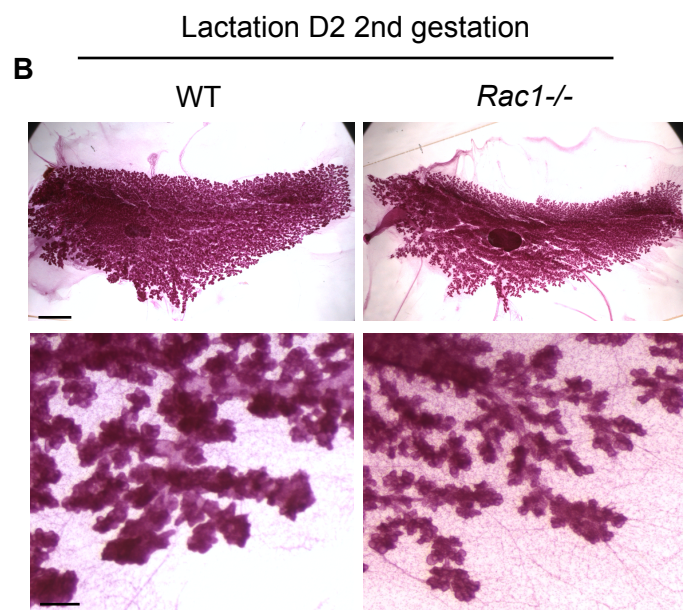

Supplement: S3 Fig — Reduced alveologenesis in the second lactation. (A, B) Carmine staining of whole-mounted mammary glands from WT and Rac1−/− mice at lactation day 1 (A) and 2 (B) following the second pregnancy reveal reduced alveologenesis. Bar: 2.8 mm (insert 0.3 mm). (PDF) [file pbio.3001583.s003.pdf]

Involution day 2

**A** Wildtype

**B** *Rac1*<sup>-/-</sup>

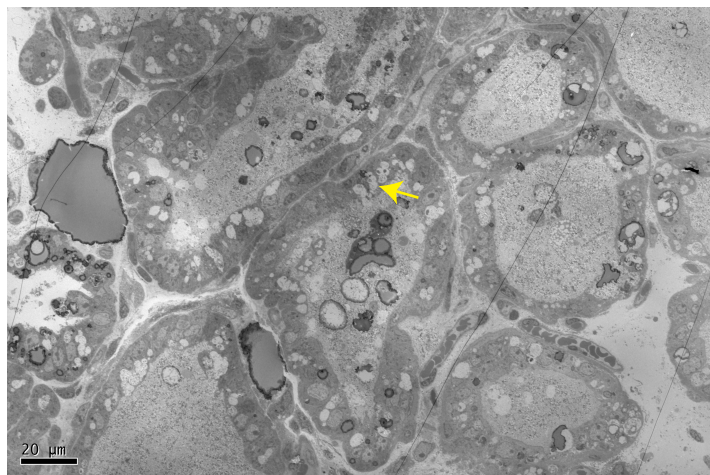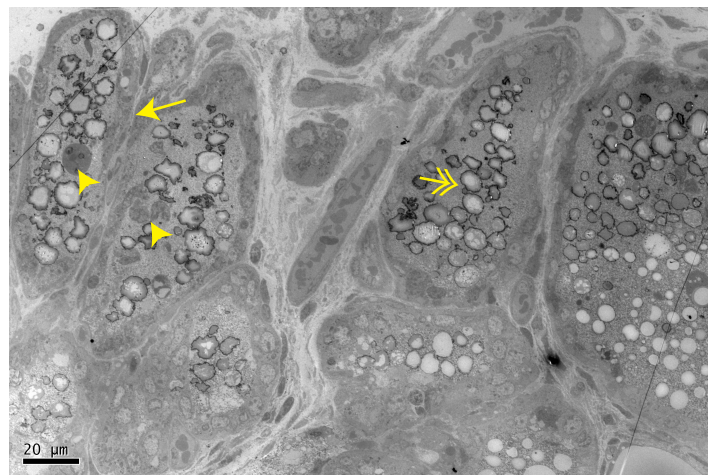

Involution day 4

**C** Wildtype

**D** *Rac1*<sup>-/-</sup>

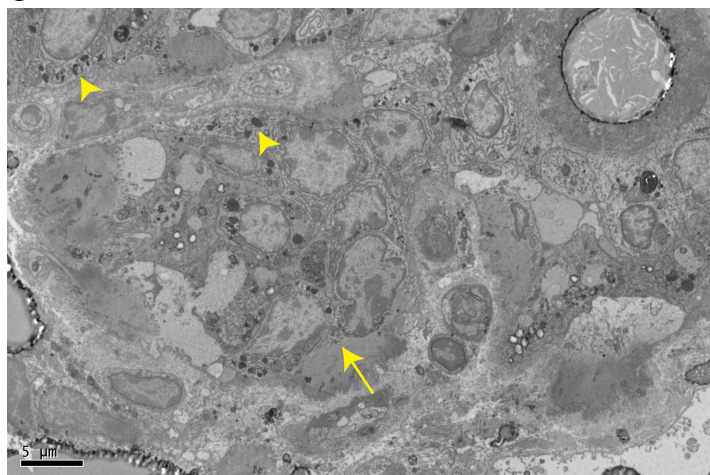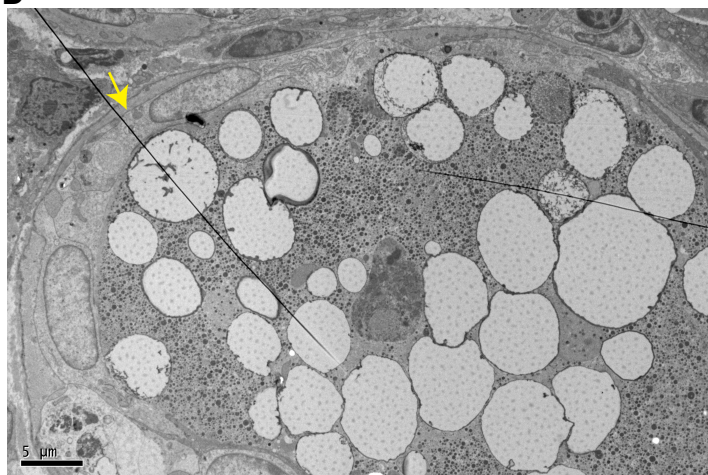

Supplement: S4 Fig — Loss of autophagosomes, phagosomes, and lysosomes in Rac1−/− glands. (A, B) Electron microscopy micrograph showing multiple alveoli from WT (A) and Rac1−/− (B) involution day 2 mammary glands. Note: Numerous autophagosome/phagosome-like structures are evident in WT alveolar epithelia (arrow; A) but not in Rac1−/− alveoli (arrow; B). Instead, Rac1−/− alveolar lumens are full of dead cells (arrowheads) and milk lipids and proteins (double arrowhead). Bar: 20 μm. (C, D) Electron microscopy micrograph showing alveoli from WT (A) and Rac1−/− (B) involution day 4 mammary glands. Note: the collapsed alveoli in WT (arrow; C) and multiple lysosomes in cells (arrowheads; C). In contrast, Rac1−/− alveoli is distended with a lack of lysosomes in cells (arrow; D). Bar: 5 μm. (PDF) [file pbio.3001583.s004.pdf]

Figure 5I

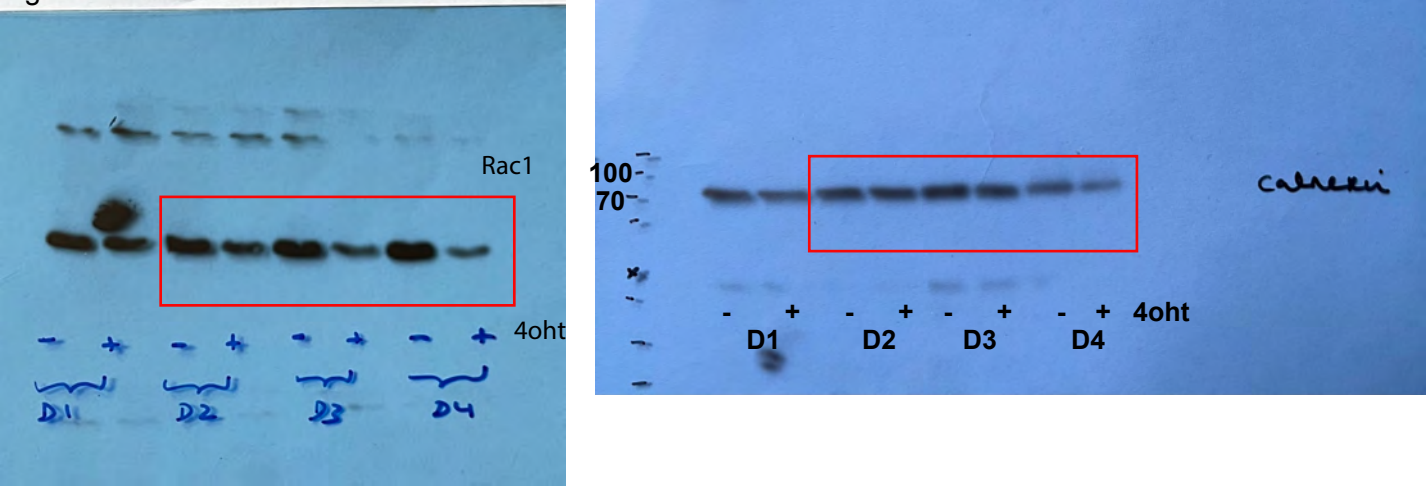

Figure 6S

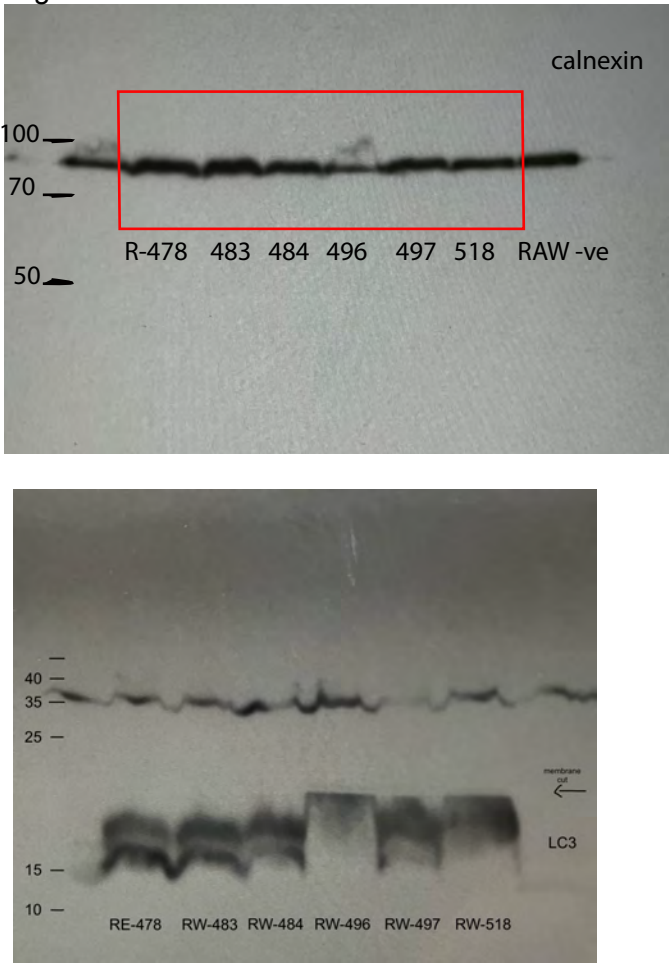

Figure S1 RAW

Supplement: S1 Raw Images — Raw images of western blots. (PDF) [file pbio.3001583.s009.pdf]
