# Supplementary material for: Rac1 controls cell turnover and reversibility of the involution process in postpartum mammary glands
Source: PLoS Biol. 2023 Jan 19;21(1):e3001583. doi: 10.1371/journal.pbio.3001583 (PMC9851507; doi:10.1371/journal.pbio.3001583)
Supplement: S1 Table — List of lysosomal genes down-regulated in Rac1−/− glands. Microarray gene expression data from WT and Rac1−/− mammary glands, showing list of lysosomal genes down-regulated in transgenics, n = 3 mice were used per condition. (PDF) [file pbio.3001583.s005.pdf]

Gene	Gene description	FC	P-Value
Ctsd	cathepsin D	-1.72	2.44E-02
Ctsb	cathepsin B	-2.51	2.75E-02
Ctsa	cathepsin A	-2.50	2.79E-02
Ctsh	cathepsin H	-1.97	6.70E-02
Tpp1	tripeptidyl peptidase I	-2.52	4.90E-03
Naga	N-acetyl galactosaminidase, alpha	-1.56	2.74E-02
Glb1	galactosidase, beta 1	-1.49	3.99E-02
Galc	galactosylceramidase	-1.48	4.84E-02
Manba	mannosidase, beta A, lysosomal	-1.70	5.50E-02
Neu1	neuraminidase 1	-2.50	4.90E-02
Gns	glucosamine (N-acetyl)-6-sulfatase	-1.55	2.69E-02
Ids	iduronate 2-sulfatase	-1.73	2.82E-02
Lipa	lysosomal acid lipase A	-4.66	3.28E-03
Dnase2a	deoxyribonuclease II alpha	-2.14	5.29E-02
Ppt1	palmitoyl-protein thioesterase 1	-2.40	5.43E-02
Psap	prosaposin	-1.82	5.36E-02
Sort1	sortilin 1	-2.07	3.79E-03

S1 Table
