# Supplementary material for: Rac1 controls cell turnover and reversibility of the involution process in postpartum mammary glands
Source: PLoS Biol. 2023 Jan 19;21(1):e3001583. doi: 10.1371/journal.pbio.3001583 (PMC9851507; doi:10.1371/journal.pbio.3001583)
Supplement: S2 Table — (PDF) [file pbio.3001583.s006.pdf]

S2 Table: Antibody information

ANTIBODY	SOURCE	IF	IB
Calnexin	Bioquote SPA-#SPC-108A/B		1;4000
Cleaved caspase-3	Cell Signalling Technology #9661	1;100	1;50
Cytokeratin 8/18	Progen #GP11	1;200	
F4:80 macrophage	Serotec #MCA497GA	1;200	
Green fluorescent protein	Invitrogen #A11122	1;200	1;2000
Ki67 (clone SP6) Rabbit polyclonal	Acris #DRM004	1;200	
Ki-67 mouse monoclonal antibody	Fisher Scientific UK Ltd #15367074	0.180555556	
LC3B antibody	Cell Signalling Technology #2775S	1;200	1;1000
Rac1 (clone 23A8)	Merck Millipore #05-389		1;1000
Smooth muscle actin (clone 1A4)	Sigma #A2547	1;500	
Bromodeoxyuridine/BrdU (BU20a)	BIO-TECHNE LTD (Novus Bio) #NBP2-32922-0.	0.180555556	
